# Abundance, distribution and potential impact of transposable elements in the genome of *Mycosphaerella fijiensis*

**DOI:** 10.1186/1471-2164-13-720

**Published:** 2012-12-22

**Authors:** Mateus F Santana, José CF Silva, Aline D Batista, Lílian E Ribeiro, Gilvan F da Silva, Elza F de Araújo, Marisa V de Queiroz

**Affiliations:** 1 Present address: Laboratório de Genética Molecular e de Microrganismo, Universidade Federal de Viçosa, Viçosa, Brazil; 2 Present address: Diretoria de Tecnologia da Informação, Universidade Federal de Viçosa, Viçosa, Brazil; 3 Present address: Embrapa Amazônia Ocidental-CPAA, Manaus, Brazil

**Keywords:** *Mycosphaerella fijiensis*, Transposable elements, RIP, Genome

## Abstract

**Background:**

*Mycosphaerella fijiensis* is a ascomycete that causes Black Sigatoka in bananas. Recently, the *M. fijiensis* genome was sequenced. Repetitive sequences are ubiquitous components of fungal genomes. In most genomic analyses, repetitive sequences are associated with transposable elements (TEs). TEs are dispersed repetitive DNA sequences found in a host genome. These elements have the ability to move from one location to another within the genome, and their insertion can cause a wide spectrum of mutations in their hosts. Some of the deleterious effects of TEs may be due to ectopic recombination among TEs of the same family. In addition, some transposons are physically linked to genes and can control their expression. To prevent possible damage caused by the presence of TEs in the genome, some fungi possess TE-silencing mechanisms, such as RIP (Repeat Induced Point mutation). In this study, the abundance, distribution and potential impact of TEs in the genome of *M. fijiensis* were investigated.

**Results:**

A total of 613 *LTR-Gypsy* and 27 *LTR-Copia* complete elements of the class I were detected. Among the class II elements, a total of 28 *Mariner*, five *Mutator* and one *Harbinger* complete elements were identified. The results of this study indicate that transposons were and are important ectopic recombination sites. A distribution analysis of a transposable element from each class of the *M. fijiensis* isolates revealed variable hybridization profiles, indicating the activity of these elements. Several genes encoding proteins involved in important metabolic pathways and with potential correlation to pathogenicity systems were identified upstream and downstream of transposable elements. A comparison of the sequences from different transposon groups suggested the action of the RIP silencing mechanism in the genome of this microorganism.

**Conclusions:**

The analysis of TEs in *M. fijiensis* suggests that TEs play an important role in the evolution of this organism because the activity of these elements, as well as the rearrangements caused by ectopic recombination, can result in deletion, duplication, inversion and translocation. Some of these changes can potentially modify gene structure or expression and, thus, facilitate the emergence of new strains of this pathogen.

## Background

*Mycosphaerella* is a large genus of plant pathogenic fungi, composed of more than 3,000 species [1]. One of the most important species is *Mycosphaerella fijiensis* Morelet [2] anamorphic *Paracercospora fijiensis*), a heterothallic ascomycete that causes Black Sigatoka in bananas. This disease was first reported in Fiji, an archipelago located in the southeast Pacific Ocean. In Latin America, this disease was first reported in 1972 [3]. Black Sigatoka results in severe economic losses due to its high capacity for destruction, representing a major social and economic problem, especially in underdeveloped countries where bananas are cultivated and used as a major food source [4].

Black Sigatoka can lead to production losses of 35-100% [4,5] and must be strictly controlled using costly fungicides [6]. The frequent and heavy use of fungicides can lead to the emergence of organisms that are resistant to the active compounds, as observed in Central America in the case of strobilurin fungicides [7]. Research projects using experimental hybrids are being performed in attempts to generate plants that are genetically resistant to *M. fijiensis*[8]. However, the high genetic diversity found in *M. fijiensis*[9,10] may represent an obstacle to the development of resistant plants because resistance may be quickly superseded.

Recently, the *M. fijiensis* genome was sequenced and became available on the Joint Genome Institute website (http://www.jgi.doe.gov/). The genome is approximately 74.1 Mb long, and half is estimated to be formed by repetitive element sequences [11]. Repetitive sequences are ubiquitous components of fungal genomes. In most genomic analyses, repetitive sequences are associated with transposable elements (TEs) [12-14].

Transposable elements can be hierarchically classified by class, subclass, order, superfamily, family and subfamily. There are two classes of TEs that differ in the presence or absence of an intermediate RNA. In class I TEs, the DNA is synthesized from a single RNA transposon copy via reverse transcriptase and is then able to insert itself elsewhere in the genome. In class II TEs, direct excision occurs, followed by integration into the genome [15].

All class I TEs transpose via an intermediate RNA that is transcribed from a single copy of the genome and produces a cDNA via reverse transcription, which is encoded by the element itself. Each complete transposition cycle produces a new copy. Consequently, retrotransposons are often the major contributors to the repetitive fraction in the genome. Retrotransposons have two major subclasses, the LTR (Long Terminal Repeat) retrotransposons and the non-LTR retrotransposons (LINEs, Long Interspersed Nuclear Elements, and SINEs, Short Interspersed Nuclear Elements), which are distinguished mainly by the respective presence or absence of LTRs at their ends. Furthermore, groups of non-autonomous TEs lack one or more of the genes essential for transposition, including MITEs (Miniature Inverted-repeat Terminal Elements) for class II, SINEs for non-LTR retrotransposons, and TRIM retrotransposons (Terminal-repeat Retrotransposon In Miniature) and LARDs (Large Retrotransposon Derivates) for LTR retrotransposons [16]. The LTR retrotransposons are prevalent in eukaryotes and contain direct-repeat sequences flanking a coding region. These retrotransposons vary in size, reaching up to 25 kb. They typically contain so-called *gag* and *pol* ORFs. The *gag* region encodes structural proteins that form a virus-like particle (capsid protein). Occasionally, the retrotransposons can also contain ORFs of unknown function. The *pol* region encodes a protease, a reverse transcriptase, an RNase and an integrase [17]. The two main superfamilies of LTR retrotransposons are *Gypsy* and *Copia*, which differ in the order of the regions that encode the reverse transcriptase and the integrase within the *pol* region [18].

Class II TEs can be divided into two subclasses. Subclass 1 comprises the TEs that are transposed by integration and excision mechanisms, in which both strands of DNA are cleaved during excision, whereas subclass 2 consists of TEs that duplicate before insertion. Subclass 1 contains two orders; the most well known is the TIR (Terminal Inverted Repeated) order. This order contains nine superfamilies: *Tc1-Mariner, Mutator, hAT, Merlin, Transib, P, PIF/Harbinger, CACTA* and *Crypton.* Subclass 2 has two orders: *Helitron* and *Maverick*[15].

The effect of TE insertion depends on the location where it occurs in the genome (e.g., exon, intron or promoter). However, few alterations are caused by a transposition event because deleterious mutations are preferentially eliminated. Thus, some of the deleterious effects of TEs may be due to ectopic recombination among TEs of the same family. To prevent possible damage caused by the presence of TEs in the genome, some fungi possess TE-silencing mechanisms, such as RIP (Repeat Induced Point mutation). RIP is a gene silencing mechanism that leads to the mutation of repeated DNA sequences during the *Neurospora crassa* sexual cycle (Selker, 1990). In general, RIP induces G:C-to-A:T mutations in duplicated DNA sequences that are longer than 400 bp and share more than 80% identity [19]. Recently, RIP has been described in a wide range of fungi belonging to different classes [11]. In specific cases, such as in Pucciniomycotina, the process and target site of hypermutation are conserved [20].

Excluding deleterious insertions, the mutational activity of TEs may promote genetic diversity and speed up the adaptation process. In addition, some transposons are physically linked to genes and can control their expression [21]. Recently, Li et al. [22] showed that many miRNAs are derived from TEs and that the incorporation of these cognate TEs into the conserved domains of genes that encode proteins may lead to their integration into regulatory networks via miRNA.

Thus, given the potential importance of transposons in the evolution of *M. fijiensis,* the present study describes an analysis of TEs to characterize the main class I and class II elements present in the genome of *M. fijiensis* and the possible impacts of their presence in the genome of the fungus that causes Black Sigatoka.

## Results

### Analysis of transposable elements in the genome of *M. fijiensis*

**Figure 1:**
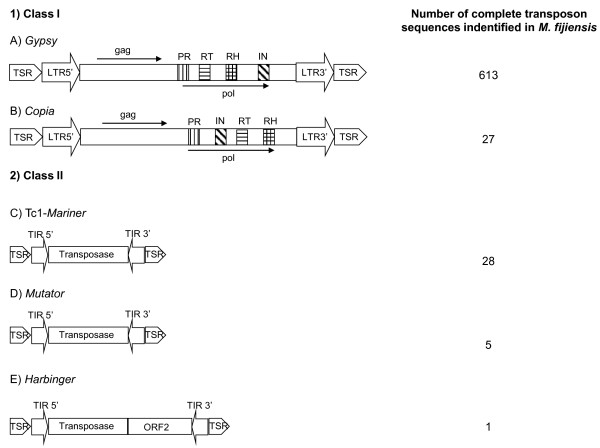
**Basic structure of the major complete transposable elements found in the genome of *M. fijiensis*.** In 1, the class I representatives are depicted as follows: *LTR-Gypsy* and *LTR-Copia* with their respective coding regions as described in the literature. The *pol* region contains the PR (protease), RT (reverse transcriptase), RH (RNase H) and IN (integrase) domains. In 2, the class II representatives are presented as follows: *Tc1-Mariner*, *Mutator* and *Harbinger*. The LTRs (Long Terminal Repeats) are indicated by wide arrows. The TIRs (Terminal Inverted Repeats) are indicated by small arrows. Each element is flanked by the insertion site or Target Site Repeat (TSR).

Using a combination of bioinformatics analyses and manual inspections, we have identified 11.7% of the sequenced genome of *M. fijiensis* as corresponding to TEs, of which 61% are related to complete copies of TEs and the other remaining 39% are degenerate copies (Table 1). Approximately 86% of the sequences identified have identity with LTR-Gypsy elements. Due to the number of accumulated mutations, very degenerate sequences may have no role in the regulation of genes and, because of decreased homology between the sequences, may not represent targets for ectopic recombination. These considerations drove us to search for complete transposable elements because such elements contain copies less affected by mutations and they can have a real impact on the evolution of this pathogen. A total of 613 *LTR-Gypsy* and 27 *LTR-Copia* elements belonging to class I were identified. Twenty-eight *Mariner*, five *Mutator* and one *Harbinger* class II elements were also identified. Together, these TEs represent approximately 5.3 Mb of the genome, corresponding to 7.1% of the sequenced genome. The structures of the main TEs identified are presented in Figure 1. Upon analysis of the genes encoding proteins related to transposition, only three *LTR-Copia* elements were identified as being potentially active in the genome. The other TEs contained multiple stop codons within the sequences encoding the proteins responsible for transposition.

**Table 1:** **Sequences of transposons identified in the genome of *M. fijiensis***

**Repetitive element**	**Number of remaining degenerate copies**	**Number of complete TEs**	**Percentage in the genome**
**Class I**	**3,508**	**640**	**11.45**%
SINE: Penelope	29	-	<0.00%
LINEs	49	-	0.01%
LTR elements: *Copia Gypsy*	3,430	640	11.44%
	251	27	0.33%
	3,044	613	10.05%
Solo-LTRs	135	-	0.05%
**Class II**	**85**	**34**	**0.17**%
*Hobo-Activator*	9	-	<0.00%
*Mariner*	59	28	0.13%
*Mutator*	12	5	0.03%
*Harbinger*	5	1	<0.00%
**Unclassified**	**56**	**-**	**<0.00**%
**Total Elements**	**3,649**	**674**	**11.7**%

The *LTR-Gypsy* elements, the main representatives in the genome of *M. fijiensis*, vary in size and are on average 6,000 to 20,000 bp. These elements contain direct LTRs containing from a few hundred bp to over 1 Kb. The 5' and 3' LTR in each element typically end in inverted repeats with the consensus 5'-TG … CA-3, as found in many retrotransposons such as Gypsy/Ty3 [15]. A total of 312 insertion sites or TSRs (Target Site Repeat) were identified, with a wide variation in the TSRs of the LTR elements (Additional file 1). A total of 515 LTR elements were analyzed; the remaining 125 LTR elements manifested differences in the 5’ and 3’ insertion sequences and were not analyzed because they showed evidence of ectopic recombination in the genome. The insertion sites varied in size from four to six bp. A total of 282 insertion sites of five bp, 21 sites of four bp and three sites of six bp were found. The majority of the four-bp insertion sites were primarily related to elements of approximately 12,000 bp. The most frequently found insertion sites were: CTATA (8), TATAG (8) and ATATA (7). Among all of the insertion sites identified, 256 sites exhibited low frequency and were observed no more than twice. Regarding the class II transposons, the insertion sites were: TA (*Tc1-Mariner*), GCAGCAACC, GACTCTGGT, TCGTCTC, TCATGCCC (*Mutator*) and CTC (*Harbinger*).

### Transposable elements physically linked to coding regions or protein domains

The analysis of the regions approximately 10,000 bp upstream and downstream of each TE allowed the identification of 339 genes encoding proteins or protein domains (Additional file 2). Several genes were identified that encoded proteins related to important metabolic pathways, such as malate synthase, malate dehydrogenase, fructose-1,6-bisphosphatase, acetyl-CoA C-acyltransferase, ribose-5-phosphate isomerase A, sucrose-6-phosphate hydrolase, phosphoglycerate mutase, ATP synthase, glutaminases and glutamate cysteine ligase (Table 2).

**Table 2:** Partial list of proteins upstream and downstream of the transposons

**Scaffold**	**Transposon**	**Gene**	**Approximate distance (bp)**	**Identity (%)**	**Similarity (%)**	**Reference (GenBank)**
1	*LTR-Gypsy*	PX Domain	D: 4,100	63	78	XP_001263638.1
1	*LTR-Gypsy*	Glutaminase A	U: 7,400	65	77	XP_001930459.1
1	*LTR-Paste*	E2-ubiquitin	D: 4900	72	78	XP_001593719.1
1	*LTR-Gypsy*	Malate synthase	D: 9,000	84	91	XP_001797883.1
1	*LTR-Gypsy*	ABC transporter	D: 5,500	70	83	XP_001727592.1
1	*LTR-Gypsy*	Glutamate cysteine ligase	D: 4,300	77	86	XP_001940223.1
1	*LTR-Copia*	Serine/threonine kinase	U: 790	64	77	XP_001819711.2
1	*LTR-Gypsy*	NADPH-cytochrome P450	D: 3,950	51	68	XP_001818965.1
1	*LTR-Gypsy*	MYND Domain	D: 3,300	52	65	XP_750050.1
1	*LTR-Gypsy*	2-methylcitrate synthase	U: 3,300	83	91	XP_965076.1
1	*LTR-Gypsy*	WD40 Domain	D: 4,500	61	71	XP_002372958.1
1	*LTR-Gypsy*	Fructose-2,6-biphosphatase	U: 4,800	73	83	XP_001546391.1
2	*LTR-Gypsy*	Acetyl-CoA C-acyltransferase	D: 7,000	70	81	XP_001392657.1
2	*LTR-Gypsy*	Sugar transporter	D: 900	57	73	XP_003069717.1
2	*LTR-Gypsy*	Acetamidase	D: 3,700	58	70	XP_001940983.1
2	*LTR-Gypsy*	LaeA	D: 3,100	50	68	XP_001827612.2
2	*LTR-Gypsy*	Hsp70	D: 6,500	69	74	XP_001818154.2
2	*LTR-Gypsy*	Chitin synthase	U: 2,200	70	82	XP_003071333.1
2	*LTR-Copia*	DNA helicase	D: 1,700	54	66	XP_001824182.2
3	*LTR-Gypsy*	ATP synthase	U: 5,000	84	92	EFX00799.1
3	*LTR-Gypsy*	Proteasome Activator Subunit4	D: 1,000	59	75	XP_751700.1
4	*LTR-Gypsy*	Glucanase	D: 3,400	60	78	XP_002624797.1
5	*LTR-Gypsy*	Malate dehydrogenase	D: 2,800	66	75	XP_001931613.1
5	*LTR-Gypsy*	SNARE Domain	U: 4,600	62	77	XP_001941286.1
6	*LTR-Gypsy*	Aflatoxin B1 aldehyde reductase	D: 8,000	59	79	XP_002845070.1
6	*LTR-Gypsy*	Alcohol dehydrogenase	U: 5,500	73	82	XP_001825083.1
7	*LTR-Gypsy*	Gamma-glutamyl transpeptidase	D: 6,800	65	78	XP_001933197.1
8	*LTR-Gypsy*	Aspartate aminotransferase	U: 1,000	70	77	XP_001933414.1
9	*DNA-Mariner*	Ribose5-phosphate isomerase A	D: 800	63	79	XP_003069185.1
9	*LTR-Gypsy*	FAD Domain	U: 1,000	64	79	XP_001263972.1
10	*LTR-Gypsy*	MFS transporter	U: 5,000	63	77	XP_749221.1
10	*LTR-Gypsy*	Sucrose-6-phosphate hydrolase	D: 9,000	56	73	XP_001936697.1
10	*LTR-Gypsy*	Ribonuclease H1	D: 800	67	76	XP_001823167.2
10	*LTR-Gypsy*	Phosphoglycerate mutase	D: 7,000	65	78	ZP_08027076.1
14	*LTR-Copia*	Histone H3	U: 100	70	84	XP_760063.1

D: downstream.

U: upstream.

Additional identified genes encoded proteins that potentially exhibit strong correlations with pathogenic systems, such as ABC (ATP binding cassette) and MFS (Major Facilitator Transporter) transporters and regulatory proteins similar to LaeA and serine/threonine kinases. The analysis also identified genes that encode proteins related to detoxification (aflatoxin B1 aldehyde reductase and gamma-glutamyl transpeptidase), and multiple protein domains involved in signal transduction, including pre-mRNAs (WD40 domain), oxidoreductases (FAD domain), ubiquitilation (MYND domain), membrane proteins (PX domain), exocytosis (SNARE domain) and proteins with functions related to apoptosis and DNA repair processes (Table 2 and Additional file 2). No complete copy of a TE has been found in a silenced gene or in an intronic region.

### Evidence of RIP in the genome of *M. fijiensis*

Most of the identified TEs contained stop codons in the sequences encoding the proteins related to transposition. Only three *LTR-Copia* elements exhibited “in silico” evidence of activity, because they have high identity among the LTRs, with ORFs consistent with complete mRNA transcription. The TpA/ApT and (CpA + TpG)/(ApC + GpT) ratios were used to investigate RIP-like events in the genome of *M. fijiensis* among the TEs with more than 80% identity. Altogether, eight TE groups were identified that shared more than 80% identity among the TEs within the same group. The groups and the respective numbers of aligned sequences were Mutator (3), Mariner1 (4), Mariner2 (3), LTR-Copia (3), LTR-Gypsy1 (7), LTR-Gypsy2 (6), LTR-Gypsy3 (11) and LTR-Gypsy4 (41). RIP-like mutations were identified when the index values generated for each element group were compared with the standards for both indices used (Table 3). 

**Table 3:** **TpA/ApT and (CpA + TpG)/(ApC + GpT) ratios for transposons in the genome of *M. fijiensis***

**Transposon (Group)**	**Number of sequences**	TpA/ApT value*	(CpA + TpG)/(ApC + GpT) value*
Mutator	3	2.03	0.36
Mariner1	4	1.93	0.32
Mariner2	3	2.09	0.55
LTR-Copia	3	1.92	0.34
LTR-Gypsy1	7	2.07	0.27
LTR-Gypsy2	6	2.10	0.25
LTR-Gypsy3	11	2.06	0.23
LTR-Gypsy4	41	1.93	0.43

*Standard reference values of the RIP indices are: TpA/ApT > 0.89 and (CpA + TpG)/(ApC + GpT) < 1.03 [51].

### Hybridization profiles related to transposons of class I and II

**Figure 2:**
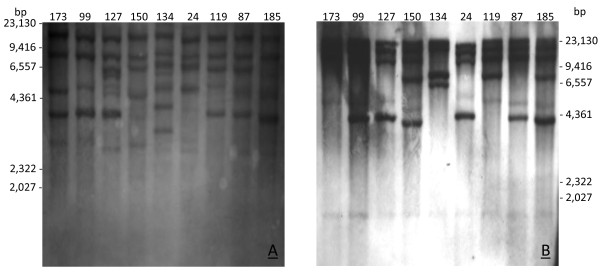
**Hybridization profiles related to class I and II elements. A)** Hybridization of isolates using a 643 bp fragment containing the reverse transcriptase gene of the *Sagui* element as a probe. **B)** Hybridization of isolates using a conserved 957 bp fragment containing part of the *Mariner* transposable element as a probe.

Two hybridizations with examples of class I and II TEs were performed in an attempt to detect possible traces of activity of these TEs in *M. fijiensis* populations. In the first hybridization, the probe used was the reverse transcriptase of the one *Sagui LTR-Copia*, which exhibited evidence of recent activity by bioinformatic analysis. This element is 4,738 bp in size, with 100% identical LTRs. The ORF encodes the conserved domains of the key proteins related to transposition, with the exception of aspartic protease, whose conserved domains are difficult to identify (Additional file 3). We have identified "in silico" four copies of this element in the genome of *M. fijiensis*. The hybridization profile of the *Sagui* element revealed copy variations among the different *M. fijiensis* isolates. Among the nine isolates analyzed, eight different hybridization profiles could be observed (Figure 2A).

In the second hybridization, the probe was constructed from the conserved regions of four *Mariner* transposons with no “in silico” evidence of activity in the sequenced genome (Additional file 4). The hybridization profile also demonstrated copy variations among the different isolates. Five different hybridization patterns could be observed among the nine isolates analyzed (Figure 2B).

## Discussion

Fungi are versatile eukaryotes that occupy different ecological niches and are responsible for several important processes, such as organic matter decomposition, symbiotic association and pathogenicity in animals and plants. This group of microorganisms is considered a model for the study of the biology and genetics of eukaryotes. Accordingly, fungi are among those groups of organisms with the largest number of genomes already sequenced or in the process of being sequenced and annotated [23,24].

The genomes of fungi contain varying numbers and sizes of repeated sequences, usually representing 3% to 10% of the sequenced genome. However, some genomes diverge from this range, such as the genome of *Ashbya gossypii*, which, surprisingly, contains no detected TEs [25], and the genome of *Tuber melanosporum*, which consists of 58% TEs [14]. In *Laccaria bicolor*, more than 215 genus-specific TEs and a large number of remaining degenerate copies were found [13]. The genome of *Mycosphaerella graminicola* contains 21.2% of repetitive sequences, and a large percentage of these sequences are in dispensable chromosomes [26]. In the present analysis, the RepeatMasker software, one of the most readily available and widely used bioinformatics tools for the detection, characterization and analysis of repetitive element sequences in the genomes of eukaryotes [27], along with the LTR-Finder and the Repeat Finder programs, determined that approximately 7% of the *M. fijiensis* genome consists of complete TEs. Using differences in the dinucleotide profile, Clutterbuck [11] estimated that approximately 50% of the genome of *M. fijiensis* is composed of repetitive elements. Compared with analysis based on anomalies in the DFD (dinucleotide frequency distribution), which have little specificity, analysis using RepeatMasker is much more specific because it uses a database (RepBase) of consensus sequences from the principal characterized transposable elements. The anomalies in the DFD may overestimate the number of transposable elements in the genome because they detect any changes in the GC content, including telomeric and centromeric sequences, material from horizontal transfer, satellite regions, supernumerary chromosomes and RIPed sequences, among others. Moreover, RIP appears to be intense in *M. fijiensis*. RIP is a mechanism that acts on not only transposable elements but also on other duplicated sequences. Thus, Clutterbuck [11] inferred a large number of repetitive sequences without specifying what percentage of these sequences are actually transposable elements. Moreover, RepeatMasker can fail to detect very degenerate copies of elements and also can miss TEs that are not represented in the database (RepBase). As the evidence suggests that the RIP process operates heavily on the genome of *M. fijiensis*, it is expected that very degenerate copies are partially identified by the program. However, due to the number of accumulated mutations, very degenerate sequences may have no role in the regulation of genes and, because of decreased homology between the sequences, may not represent targets for ectopic recombination. These considerations drove us to search for intact transposable elements because such elements contain copies less affected by mutations and they can have a real impact on the evolution of this pathogen.

In terms of the types of TEs identified, retrotransposons appear to be largely responsible for the repetitive fraction of the *M. fijiensis* genome*.* These elements were found in hundreds of copies and exhibit great family diversity. *Gypsy/Ty3* has been the main TE group identified in phytopathogenic fungi [28] and has also been widely identified in the genome of *M. fijiensis.* The class II TEs are typically ancient elements found in almost all eukaryotes; however, they are usually found in a small number of copies [15]. The best represented class II elements were those belonging to the *Tc1-Mariner* superfamily, one of the most diverse and widely distributed in nature. Another superfamily identified that occurs in various species of eukaryotes was the *Mutator* superfamily. Both superfamilies encode a transposase and are flanked by TIRs; however, they differ in relation to the insertion site. Elements of the *Tc1-Mariner* superfamily usually insert into TA sequences, while TEs of the *Mutator* superfamily have insertion sites that vary from 9 to 11 bp [15]. Finally, an element belonging to the *Harbinger* superfamily exhibited a high accumulation of mutations and did not allow for the detection of conserved domains. Elements belonging to this superfamily generally have two ORFs, one encoding a DNA binding protein and the other encoding a transposase [15].

There is strong evidence that ectopic recombination events are now or have been very intense in the genome of *M. fijiensis.* This is because, in addition to finding a large number of degenerate sequences and solo LTRs, 125 identified retrotransposons had different insertion sites flanking the 5’ and 3’ end of the same element. The presence of different insertion sites at the ends of the same TE and the presence of numerous degenerate sequences are indicative of ectopic recombination among retrotransposons. Recombination events can influence the adaptation of this species by promoting rearrangements (deletion, duplication, inversion or translocation) and chromosome breakage [12]. In *Magnaporthe grisea*, the analysis of the distribution of transposable elements in the genome has highlighted the fact that in the past there was an extensive ectopic recombination. As this organism relies on asexual propagation, recombination events can help improve the adaptation of these microorganisms because many genes that contribute to host specificity are present in regions rich in transposable elements. Thus, recombination events can lead to deletions or alterations in the structure of these genes and therefore altered expression [12]. The involvement of TEs in ectopic recombination has also been inferred in *Coprinus cinereus*[29] and *Verticillium dahliae*[30].

Possible TE activity has been identified in many sequenced fungal genomes. In *L. Bicolor*, 40 different TE families were observed, but the accumulation of mutations in the nucleotides was less than 5%, indicating that the TEs were recently active. Therefore, the potential activity of these elements could be inferred [13]. In the genome of *Fusarium oxysporum*, the potential activity of these elements has been identified in several families [31]. The analysis of coding proteins from TEs showed that only three *LTR-Copia* elements contained uninterrupted ORFs and were potentially active. The high number of stop codons identified in the TEs could be explained by the presence of efficient transposon silencing mechanisms. In fact, our results indicated RIP-like events with preferred mutations in CpG dinucleotides in both class I and II TEs. The RIP index values were highly significant when compared with the set default values and standards set in other TEs analyzed in different fungi , such as PetTra in *Penicillium chrysogenum*[32] and OPHIO3-1414 in *Ophiostoma ulmi*[33], demonstrating that this process must have been or is intense in *M. fijiensis*. Furthermore, compared to the *punt* element of *Neurospora crassa*[30,34], where RIP is considered a severe event, all of the TEs analyzed in *M. fijiensis* exhibited higher values. RIP-like events in *M. fijiensis* have also been identified by Clutterbuck [11]. However, only one transposon with three representatives was analyzed. The present study analyzed a total of 78 transposons. The existence of RIP in certain genomes can carry a high evolutionary cost, as observed in *N. crassa*, where RIP could be correlated with the absence or paucity of duplicated genes in the genome. Because gene duplication is important for the evolution of any species, the existence of RIP may have a significant impact on the genomes of several fungi [35]. However, there is also the possibility that RIP can be mild, leaving one or more copies of a gene functional, and giving rise to novel alleles [31].

The hybridization profile found for the *Sagui* element evidence the recent activity of TEs, given that a large proportion of the hybridization profiles found in different isolates were polymorphic, which can be correlated with the recent activity of the element in *M. fijiensis* populations. *Sagui* has been identified and characterized as being potentially active because it possesses complete LTRs and ORFs containing the domains of the key proteins involved in transposition. Only the aspartic proteinase domain was not detected. However, this is an expected result, given that this protein is thought to be difficult to analyze because of its low similarity and different evolution rates [32,36]. Regarding the *Mariner* element, although no traces of activity were observed in the analyzed copies, the hybridization profiles of the different isolates showed polymorphisms, consistent with active TEs in the *M. fijiensis* populations. Another explanation for the few active TEs in the analyzed genome may be the fact that in most sequenced fungi species, the genome is highly stable because it has been maintained under laboratory conditions for long periods of time. However, we must emphasize that defective or non-autonomous elements can be mobilized in trans by related active elements containing proteins with motif sequences recognized by enzymes that are essential to transposition [15,37]. Moreover, degenerate sequences can still have the ability to modify gene expression of the neighboring genes. Another important aspect is that the hybridization profile detected emphasizes the possibility of the use of such elements as molecular markers to trace the population structure of *M. fijiensis* in places where this disease has been described.

Genes encoding proteins that may be related to pathogenic mechanisms have been identified around complete TEs. Many genes for ABC and MFS transporters have been identified near TEs-rich regions. Some of these transporters have an important role as drug carriers and, therefore, provide protection to the organism against toxic products and fungicides. In plant pathogens, these transporters may be associated with multidrug resistance, virulence and altered sensitivity to fungicides [38,39]. Another gene identified near a TE encodes a protein similar to LaeA, a regulator of virulence genes and, possibly, the first antimicrobial target specific for filamentous fungal pathogens of plants and animals [40]. Similarly, TEs have been found near important genes related to the pathogenicity system in two important plant pathogens, *M. grisea* and *F. oxysporum*. At first, Khang [41] studied the gene AVR-Pita in the pertaining to avirulence gene family. These authors discovered that members of this family are associated with different types of transposable elements. The activity of these elements, as well as rearrangements caused by ectopic recombination, can potentially modify the structure or expression of AVR genes, and thus new races of the pathogen may emerge. In *F. oxysporum*, certain regions of the genome related to pathogenicity have 74% of transposable elements identified in the genome, including 95% of all DNA transposons that may be involved in gene duplication events [31].

Several genes encoding proteins involved in vital processes were found near TE-related sequences. Genes encoding proteins such as chitin synthase, involved in cell wall biogenesis, were found in regions with a high density of transposon-related sequences. Several sequences encoding serine/threonine kinase proteins have been identified. These protein domains are related to different regulatory pathways in cellular processes, such as growth, sexual/asexual development [42] and pathogenicity [43]. Our results also identified several genes near TEs encoding proteins with important roles in transcription, translation, replication, cellular respiration, nutrient and ion transport, DNA repair, ubiquitination, apoptosis and cell wall formation and stabilization as well as those involved in important metabolic pathways, such as fatty acid metabolism, pyruvate metabolism and amino acid and vitamin biosynthesis and degradation. Our results show that the insertions of transposable elements in the genome of *M. fijiensis* are probably harmless. However, the activity of the elements near important genes can potentially modify gene expression, as well as the rearrangements caused by ectopic recombination can modify gene structure.

A final relevant fact regarding the presence and maintenance of transposable elements in the genome of several species is the possible role of TEs in gene regulation. Excluding deleterious insertions, TEs may be linked to the regulation of gene expression. This is a process known as domestication and represents an example of the exaptation of TEs at the molecular level, which would explain their maintenance in the genome of several species [21]. Recently, humans miRNAs derived from TEs have been implicated in the regulation of important pathways, such as cell proliferation, chromosome segregation, mitosis and apoptosis [44]. In addition, miRNAs based on TEs may represent essential components in the maintenance of genomic stability, serving as a safeguard for genome integrity and potentially functioning as an anti-cancer defense mechanism [45]. In fungi, little is known about miRNA regulators. Transposable element domestication through miRNA-based regulation systems may be another important contribution of TEs in fungi. Therefore, further investigations into TE dynamics and their role in regulatory networks via mRNA should be performed in *M. fijiensis*, especially in light of the strong evidence reported in the present study about the organization and possible impacts of the presence of transposons in the genome of this fungus.

## Conclusions

The analysis of TEs in *M. fijiensis* suggests that TEs play an important role in the evolution of this organism because the activity of these elements, as well as the rearrangements caused by ectopic recombination, can result in deletion, duplication, inversion and translocation. Some of these changes can potentially modify gene structure or expression and, thus, facilitate the emergence of new strains of this pathogen.

The existence of RIP may have a significant impact on the genomes of *M. fijiensis* because the occurrence of RIP prevents the accumulation of transposable elements in fungi and this mechanism may also be related to the gradual divergence of duplicated genes, a process regarded as essential for the emergence of genes with new functions.

A thorough study and understanding of the role of TEs in *M. fijiensis* would allow for more comprehensive understanding of the genome organization. In addition, these TEs have low target site specificity, so it can be used for mutagenis or as molecular markers to study population and genetic diversity.

## Methods

### Identification of isolates and total DNA extraction

The isolates were provided by Embrapa Amazônia Ocidental - CPAA (Table 4), and the total DNA was extracted from the isolates according to Specht et al. [46]. 

**Table 4:** Isolates used in this study

**Collection ID**	**Origin**	**Host genotype**	**Geographical coordinates**
24Mf	Rio Preto da Eva - AM	Prata	S 02 43 040 W 59 4 515
87Mf	Caceres - MT	Grand Naine	S 16 09 147 W 57 37 914
99Mf	Iranduba - AM	Pacovan	S 03 11 633 W 60 08 392
119Mf	Caroebe - RR	Prata	S 00 47 820 W 59 25 749
127Mf	Presidente Figueiredo - AM	Carú roxa	S 02 03 335 W 59 38 652
134Mf	Atalaia do Norte - AM	Prata	S 04 22 598 W 70 10 356
150Mf	Itacoatiara - AM	Prata	S 03 03 520 W 58 50 140
173Mf	Careiro Castanho - AM	Pacovan	S 03 43 345 W 60 16 700
185Mf	Rio Branco - AC	D’angola	S 10 06 137 W 67 29 718

AM - Amazonas, Brazil; MT - Mato Grosso, Brazil; RR - Roraima, Brazil; AC - Acre, Brazil.

### Identification and classification of transposable elements

The genome of *M. fijiensis* was obtained from the Joint Genome Institute database (http://www.jgi.doe.gov/genome-projects/). The identification and classification of the repetitive element sequences in the genome of *M. fijiensis* was performed using the RepeatMasker software (A.F.A. Smit, R. Hubley & P. Green, RepeatMasker at http://repeatmasker.org). This program identifies TE copies by comparing the genomic sequences with sequences present in a previously described TE library (RepBase 16.12: http://www.girinst.org/Rpbase-Update.html) [47]. The present study used the fungal TE library (fngrep.ref). The following parameters were used for this search: “cross_match” as the search model; “slow search” to obtain a search 0-5% more sensitive than the standard; “fungi” to specify the species or group of input sequences and “alignment” to generate an output file showing the alignment. However, this software detects only genomic regions showing identity with the database sequences, and in many cases, it is not possible to find complete TEs. Thus, after the identification of sequences by RepeatMasker, approximately 10,000 bp upstream and downstream of each marking were submitted to the LTR-Finder (http://tlife.fudan.edu.cn/ltr_finder/) [46] and Repeat Finder [48] programs to find the ends of each repeating element and thereby define the complete copies of the elements. Searches for complete class I TEs were performed using LTR-Finder [49] and Repeat Finder [48] to identify LTRs (Long Terminal Repeats). The Repeat Finder software [48] was used to identify TIRs (Terminal Inverted Repeats) within the complete class II elements. Elements that do not naturally possess repeated ends were examined via BLASTN at the NCBI website (http://www.ncbi.nlm.nih.gov) to determine the presence of complete copies of these elements. An analysis of the ORFs within the coding region of each TE was performed using ExPASy (http://expasy.org/) and ORF-finder (http://www.ncbi.nlm.nih.gov/projects/gorf/).

The sequences found were classified as complete elements, active elements, and degenerate sequences. Complete elements contain sequence similarity with proteins related to transposition machinery, terminal repeats conserved, and target site duplication (TSD). Active elements are complete elements that contain intact protein domains and characteristic open reading frame (ORFs) for specific superfamily or subclass of transposons. Degenerate sequences contain sequence identity with consensus sequences from the principal characterized transposable elements (RepBase), however lack structural features or protein coding sequences related to transposition.

The insertion sites or TSR (Target Site Repeat) of the TEs were characterized by direct visualization of the sequences flanking each TE. The TEs that had diverging 5’ and 3’ insertion sequences were not assessed for TSR.

After searching for complete TEs, the regions approximately 10,000 bp upstream and downstream of each TE were analyzed using the BLASTX tool (http://www.ncbi.nlm.nih.gov/BLAST) and the RefSeq_protein (Reference Sequence Protein) database to identify protein coding sequences around the TEs. The threshold used for the identification of proteins was E-value > 10^-20^ and identity > 50%.

Potentially active elements were identified “in silico” through the presence of ORFs with protein domains that are typically required for transposition and conservation of LTRs for class I elements and TIRs for class II elements.

### Evidence of the RIP silencing mechanism

For the analysis of dinucleotides and the calculation of the RIP indices, TEs with more than 80% identity were aligned using the Mega 4 software [50]. Subsequently, the RipCal software [51] was used to calculate the TpA/ApT and (CpA + TpG)/(ApC + GpT) ratios. The TpA/ApT ratio is a simple index that measures the frequency of RIP products, TpA, with a false positive correlation due to ApT-rich regions. High TpA/ApT values indicate strong evidence of RIP. In principle, the (CpA + TpG)/(ApC + GpT) ratio is similar to the TpA/ApT, but it measures the depletion of the RIP targets, CpA and TpG. In this case, low (CpA + TpG)/(ApC + GpT) values are strongly indicative of RIP. The standard reference values of the RIP index are: TpA/ApT > 0.89 and (CpA + TpG)/(ApC + GpT) < 1.03 [51].

### Integration profile analysis

To analyze the integration profile of a potentially active *LTR-Copia* retrotransposon, the following primer pair was used: RT-Copia1F (CGATACTCGGAAGGTTTCGT) and RT-Copia1R (ACTACCGAACGGACAAATCG), which amplified a region containing the reverse transcriptase. The 643 bp amplified sequence was used as a probe (Additional file 3). This TE can be found in the Scaffold 20 and was named *Sagui.* Another probe was generated from the conserved regions of four *Mariner* elements representative of class II. The sequence was approximately 957 bp and contained part of the transposase gene (Additional file 4). To synthesize the probe, the following primer pair was used: MF2mar2F (CGGTGTTTCCGAGCGAAGTTA) and MF2mar2R (AGGAAAGCGGAAGTCGAAGAA). The PCR reactions were performed in a PTC-100 Thermal Cycler (MJ Research) programmed to perform an initial denaturing step of 3 minutes at 95°C, followed by 31 cycles of 30 seconds at 95°C, 30 seconds at 58°C for the *Mariner* probe or 50°C for the *Sagui* probe, 1 minute at 72°C and a final extension step of 10 minutes at 72°C. The Roche PCR DIG Probe Synthesis Kit was used to label the probe according to the manufacturer’s recommendations.

The total DNA from the isolates was cleaved by the restriction enzyme *EcoR*I, which was chosen because it does not cleave the DNA sequences used as probes. The cleaved fragments were separated by electrophoresis on 0.8% agarose gels. The DNA fragments were transferred from the agarose gel to a nylon membrane according to Sambrook et al. [52]. The hybridizations were performed at 42% overnight. The Roche Detection Starter Kit II was used according to the manufacturer’s recommendations.

## Competing interests

The authors declare that they have no competing interests.

## Authors’ contributions

This study was conceptualized planned by MVQ and GFS. MFS performed the laboratory experiments, “in silico” and preparation of the manuscript. MVQ coordinated and guided the research, assisted with data analysis and interpretation and helped to prepare the manuscript. EFA and GFS assisted with the manuscript preparation and were co-mentors for MFS. JCFS, ADB and LER assisted with data analysis. All authors have read and approved the final manuscript.

## Supplementary Material

Additional file 1 **Flanking sequences of full copies of the transposable elements.** The table contains the variation in the TSRs (Target Site Repeat) of the transposable elements found. Click here for file

Additional file 2 **Sequences coding proteins downstream and upstream of full copies of the transposable elements.** The table contains an analysis of the regions approximately 10,000 bp upstream and downstream of each transposable element with identification of 339 genes encoding proteins or protein domains. Click here for file

Additional file 3 **Basic structure of the *Sagui *transposable element.** The figure contains the nucleotide sequence, the amino acid sequence, and the sequence used as a probe for the detection of *Sagui* in *M. fijiensis* populations. Click here for file

Additional file 4 **Alignment of four conservative sequences of *Mariner *elements used as probe.** The figure contains the alignment of Fasta sequences used to primer design to obtain the probe for the detection of *Mariner* elements in *M. fijiensis* populations. Click here for file
