# Supplementary material for: Abundance, distribution and potential impact of transposable elements in the genome of Mycosphaerella fijiensis
Source: BMC Genomics. 2012 Dec 22;13:720. doi: 10.1186/1471-2164-13-720 (PMC3562529; doi:10.1186/1471-2164-13-720)
Supplement: Additional file 1 — Flanking sequences of full copies of the transposable elements. The table contains the variation in the TSRs (Target Site Repeat) of the transposable elements found. [file 1471-2164-13-720-S1.docx]

**Table1 Flanking sequences of full copies of the transposable elements.**

| **Element** | **Flanking sequences** | **Frequency** |
| --- | --- | --- |
| *LTR-Copia* | GGTTG | 2 |
| *LTR-Copia* | CGTTG | 1 |
| *LTR-Copia* | CTGCT | 2 |
| *LTR-Copia* | GTACT | 1 |
| *LTR-Copia* | GATAT | 2 |
| *LTR-Copia* | GTGTA | 1 |
| *LTR-Copia* | GCCT | 1 |
| *LTR-Copia* | GCCAT | 1 |
| *LTR-Copia* | TGTTT | 2 |
| *LTR-Copia* | CGAAT | 1 |
| *LTR-Copia* | AGTG | 1 |
| *LTR-Copia* | TCCAG | 1 |
| *LTR-Copia* | GTCGA | 2 |
| *LTR-Copia* | CTATA | 8 |
| *LTR-Copia* | GGTAG | 3 |
| *LTR-Copia* | AGGAG | 1 |
| *LTR-Copia* | CAGTT | 1 |
| *LTR-Copia* | CTTCC | 1 |
| *LTR-Copia* | TCGAT | 1 |
| *LTR-Copia* | ATATA | 7 |
| *LTR-Gypsy* | CTTAG | 4 |
| *LTR-Gypsy* | ACCTA | 2 |
| *LTR-Gypsy* | ATAAT | 3 |
| *LTR-Gypsy* | TAAC | 1 |
| *LTR-Gypsy* | TAAGC | 1 |
| *LTR-Gypsy* | GCTAA | 2 |
| *LTR-Gypsy* | GGCTC | 1 |
| *LTR-Gypsy* | GACTC | 1 |
| *LTR-Gypsy* | TTATA | 4 |
| *LTR-Gypsy* | AATAT | 5 |
| *LTR-Gypsy* | GTTTT | 4 |
| *LTR-Gypsy* | AAGGC | 2 |
| *LTR-Gypsy* | CTAAG | 5 |
| *LTR-Gypsy* | GGTCT | 2 |
| *LTR-Gypsy* | AGAG | 1 |
| *LTR-Gypsy* | GCTAG | 4 |
| *LTR-Gypsy* | GCCGC | 1 |
| *LTR-Gypsy* | ATACG | 2 |
| *LTR-Gypsy* | ATTAG | 6 |
| *LTR-Gypsy* | CCCTT | 1 |
| *LTR-Gypsy* | GTATT | 2 |
| *LTR-Gypsy* | GCTTC | 1 |
| *LTR-Gypsy* | TATAG | 8 |
| *LTR-Gypsy* | AAGAG | 3 |
| *LTR-Gypsy* | ATTAT | 4 |
| *LTR-Gypsy* | AGTAT | 1 |
| *LTR-Gypsy* | TTCTA | 1 |
| *LTR-Gypsy* | CCCTA | 2 |
| *LTR-Gypsy* | AAGAA | 1 |
| *LTR-Gypsy* | ATGGT | 1 |
| *LTR-Gypsy* | CCTAT | 5 |
| *LTR-Gypsy* | TTATT | 1 |
| *LTR-Gypsy* | CTATT | 5 |
| *LTR-Gypsy* | ATAC | 1 |
| *LTR-Gypsy* | TATAC | 4 |
| *LTR-Gypsy* | CTCCT | 2 |
| *LTR-Gypsy* | CGAAG | 1 |
| *LTR-Gypsy* | TATA | 1 |
| *LTR-Gypsy* | TAGTT | 1 |
| *LTR-Gypsy* | CTAGA | 2 |
| *LTR-Gypsy* | TAGCC | 1 |
| *LTR-Gypsy* | TATAT | 3 |
| *LTR-Gypsy* | TCGAC | 1 |
| *LTR-Gypsy* | CCTTC | 3 |
| *LTR-Gypsy* | CTAGC | 2 |
| *LTR-Gypsy* | ACTAT | 5 |
| *LTR-Gypsy* | GTTAT | 2 |
| *LTR-Gypsy* | GGGAA | 1 |
| *LTR-Gypsy* | CTCTT | 2 |
| *LTR-Gypsy* | TACCG | 1 |
| *LTR-Gypsy* | AGTCT | 1 |
| *LTR-Gypsy* | CGACC | 2 |
| *LTR-Gypsy* | TTAAT | 4 |
| *LTR-Gypsy* | CGCG | 1 |
| *LTR-Gypsy* | CCGAG | 4 |
| *LTR-Gypsy* | GATAG | 5 |
| *LTR-Gypsy* | CTTCT | 3 |
| *LTR-Gypsy* | TACTTA | 1 |
| *LTR-Gypsy* | ATAG | 5 |
| *LTR-Gypsy* | CTTTA | 4 |
| *LTR-Gypsy* | GATTA | 1 |
| *LTR-Gypsy* | TATAA | 1 |
| *LTR-Gypsy* | CTACC | 5 |
| *LTR-Gypsy* | TAAAT | 2 |
| *LTR-Gypsy* | CTTAA | 2 |
| *LTR-Gypsy* | TTGTC | 1 |
| *LTR-Gypsy* | GAGAT | 3 |
| *LTR-Gypsy* | ACTCG | 3 |
| *LTR-Gypsy* | CTAT | 2 |
| *LTR-Gypsy* | ATAAC | 3 |
| *LTR-Gypsy* | CTCTA | 3 |
| *LTR-Gypsy* | ATAAG | 4 |
| *LTR-Gypsy* | GTAG | 4 |
| *LTR-Gypsy* | CTAAA | 4 |
| *LTR-Gypsy* | ATGTT | 1 |
| *LTR-Gypsy* | CTAG | 5 |
| *LTR-Gypsy* | ACTAA | 2 |
| *LTR-Gypsy* | GAGGG | 1 |
| *LTR-Gypsy* | TAAGA | 1 |
| *LTR-Gypsy* | CAAAT | 1 |
| *LTR-Gypsy* | ATATT | 3 |
| *LTR-Gypsy* | ATCTA | 1 |
| *LTR-Gypsy* | ACGT | 1 |
| *LTR-Gypsy* | CTAC | 1 |
| *LTR-Gypsy* | GTAT | 1 |
| *LTR-Gypsy* | TAGAG | 2 |
| *LTR-Gypsy* | TTAT | 1 |
| *LTR-Gypsy* | TTAG | 1 |
| *LTR-Gypsy* | CTAGT | 3 |
| *LTR-Gypsy* | CTAA | 1 |
| *LTR-Gypsy* | AGGAT | 1 |
| *LTR-Gypsy* | GCAG | 1 |
| *LTR-Gypsy* | TTTAG | 1 |
| *LTR-Gypsy* | CTTTT | 1 |
| *LTR-Gypsy* | AGGGG | 2 |
| *LTR-Gypsy* | CAATA | 1 |
| *LTR-Gypsy* | TAACT | 1 |
| *LTR-Gypsy* | TACTC | 2 |
| *LTR-Gypsy* | GTAGT | 5 |
| *LTR-Gypsy* | ATAT | 1 |
| *LTR-Gypsy* | CCGTC | 1 |
| *LTR-Gypsy* | AAAGA | 2 |
| *LTR-Gypsy* | ATAGC | 3 |
| *LTR-Gypsy* | CCTAG | 4 |
| *LTR-Gypsy* | CTTAC | 2 |
| *LTR-Gypsy* | AAGTA | 3 |
| *LTR-Gypsy* | TATTA | 1 |
| *LTR-Gypsy* | TAAAG | 2 |
| *LTR-Gypsy* | ACTAC | 2 |
| *LTR-Gypsy* | TCTTA | 1 |
| *LTR-Gypsy* | GACGC | 1 |
| *LTR-Gypsy* | GCTAT | 3 |
| *LTR-Gypsy* | CTTGG | 1 |
| *LTR-Gypsy* | GCCGG | 1 |
| *LTR-Gypsy* | CGTAG | 2 |
| *LTR-Gypsy* | AGCTA | 2 |
| *LTR-Gypsy* | TTTTT | 1 |
| *LTR-Gypsy* | GCTTT | 1 |
| *LTR-Gypsy* | GCGGG | 2 |
| *LTR-Gypsy* | GAAGC | 2 |
| *LTR-Gypsy* | TCTAG | 2 |
| *LTR-Gypsy* | TTCCT | 1 |
| *LTR-Gypsy* | TCGTG | 1 |
| *LTR-Gypsy* | ACGAG | 4 |
| *LTR-Gypsy* | AATAG | 1 |
| *LTR-Gypsy* | GCTTA | 2 |
| *LTR-Gypsy* | ATAGG | 1 |
| *LTR-Gypsy* | CTAAT | 1 |
| *LTR-Gypsy* | GTAGC | 1 |
| *LTR-Gypsy* | CCTCT | 1 |
| *LTR-Gypsy* | GGCAG | 1 |
| *LTR-Gypsy* | TAGTC | 1 |
| *LTR-Gypsy* | TAGAT | 2 |
| *LTR-Gypsy* | TCTAT | 2 |
| *LTR-Gypsy* | AATTG | 1 |
| *LTR-Gypsy* | TTAAC | 1 |
| *LTR-Gypsy* | AATTA | 1 |
| *LTR-Gypsy* | GAATC | 2 |
| *LTR-Gypsy* | GCCTA | 2 |
| *LTR-Gypsy* | CAGAG | 1 |
| *LTR-Gypsy* | CTACG | 1 |
| *LTR-Gypsy* | CTACT | 2 |
| *LTR-Gypsy* | AAAAG | 2 |
| *LTR-Gypsy* | TTAGG | 1 |
| *LTR-Gypsy* | TACCT | 1 |
| *LTR-Gypsy* | AAGAC | 1 |
| *LTR-Gypsy* | AGAAG | 3 |
| *LTR-Gypsy* | ACGAC | 3 |
| *LTR-Gypsy* | CGTTT | 2 |
| *LTR-Gypsy* | AGCAC | 2 |
| *LTR-Gypsy* | ACTAG | 1 |
| *LTR-Gypsy* | TAATA | 1 |
| *LTR-Gypsy* | TGTTC | 1 |
| *LTR-Gypsy* | GGCGT | 1 |
| *LTR-Gypsy* | GCGGA | 2 |
| *LTR-Gypsy* | ATGTC | 2 |
| *LTR-Gypsy* | TACGG | 1 |
| *LTR-Gypsy* | AGAGC | 1 |
| *LTR-Gypsy* | CCCGC | 1 |
| *LTR-Gypsy* | CGCGG | 1 |
| *LTR-Gypsy* | CGATC | 1 |
| *LTR-Gypsy* | AACGT | 1 |
| *LTR-Gypsy* | GGTAC | 1 |
| *LTR-Gypsy* | TCTA | 1 |
| *LTR-Gypsy* | TTTTC | 2 |
| *LTR-Gypsy* | CCGTA | 1 |
| *LTR-Gypsy* | ATAGT | 3 |
| *LTR-Gypsy* | AAGCG | 1 |
| *LTR-Gypsy* | GGTAA | 1 |
| *LTR-Gypsy* | TCGCA | 1 |
| *LTR-Gypsy* | TAGGG | 1 |
| *LTR-Gypsy* | CCCTC | 3 |
| *LTR-Gypsy* | TCCGA | 1 |
| *LTR-Gypsy* | ACGCT | 2 |
| *LTR-Gypsy* | GTATC | 3 |
| *LTR-Gypsy* | GCCTT | 2 |
| *LTR-Gypsy* | TAGGA | 1 |
| *LTR-Gypsy* | CTATC | 2 |
| *LTR-Gypsy* | ACGAT | 1 |
| *LTR-Gypsy* | AGAAC | 1 |
| *LTR-Gypsy* | AGGTA | 1 |
| *LTR-Gypsy* | GTAGG | 1 |
| *LTR-Gypsy* | GTAAG | 1 |
| *LTR-Gypsy* | TTCTT | 2 |
| *LTR-Gypsy* | CGCCT | 1 |
| *LTR-Gypsy* | GTAAT | 1 |
| *LTR-Gypsy* | GGAGA | 1 |
| *LTR-Gypsy* | ATAGA | 2 |
| *LTR-Gypsy* | GTTCT | 1 |
| *LTR-Gypsy* | GTCCG | 1 |
| *LTR-Gypsy* | CTCCG | 1 |
| *LTR-Gypsy* | AATTC | 1 |
| *LTR-Gypsy* | GAGAG | 1 |
| *LTR-Gypsy* | AGGCG | 1 |
| *LTR-Gypsy* | GGTAT | 1 |
| *LTR-Gypsy* | ACGGC | 2 |
| *LTR-Gypsy* | CGGGA | 1 |
| *LTR-Gypsy* | GTTA | 1 |
| *LTR-Gypsy* | TCGAG | 1 |
| *LTR-Gypsy* | CCTAC | 1 |
| *LTR-Gypsy* | ATATC | 1 |
| *LTR-Gypsy* | TTAAG | 3 |
| *LTR-Gypsy* | ATTTC | 1 |
| *LTR-Gypsy* | AAGAT | 1 |
| *LTR-Gypsy* | ACGGA | 1 |
| *LTR-Gypsy* | TAATC | 1 |
| *LTR-Gypsy* | GGAAT | 1 |
| *LTR-Gypsy* | GAAAC | 1 |
| *LTR-Gypsy* | AATAA | 1 |
| *LTR-Gypsy* | AACCG | 1 |
| *LTR-Gypsy* | CTTGT | 1 |
| *LTR-Gypsy* | GAAGT | 1 |
| *LTR-Gypsy* | AGTTA | 1 |
| *LTR-Gypsy* | CTTAT | 3 |
| *LTR-Gypsy* | GAGGT | 2 |
| *LTR-Gypsy* | ACTCT | 1 |
| *LTR-Gypsy* | TACGT | 1 |
| *LTR-Gypsy* | GTAAC | 1 |
| *LTR-Gypsy* | AGACC | 2 |
| *LTR-Gypsy* | CGTAT | 1 |
| *LTR-Gypsy* | AGTAG | 2 |
| *LTR-Gypsy* | AGCAA | 1 |
| *LTR-Gypsy* | AGTCG | 1 |
| *LTR-Gypsy* | CGTCG | 1 |
| *LTR-Gypsy* | TCGGG | 1 |
| *LTR-Gypsy* | AGTAA | 1 |
| *LTR-Gypsy* | CGCTA | 1 |
| *LTR-Gypsy* | ATTCG | 1 |
| *LTR-Gypsy* | CCTTA | 1 |
| *LTR-Gypsy* | TAGAGC | 1 |
| *LTR-Gypsy* | CTCGA | 1 |
| *LTR-Gypsy* | GAACT | 1 |
| *LTR-Gypsy* | TGCTC | 1 |
| *LTR-Gypsy* | AAAGC | 1 |
| *LTR-Gypsy* | GTTTA | 1 |
| *LTR-Gypsy* | AAATG | 1 |
| *LTR-Gypsy* | AGATA | 1 |
| *LTR-Gypsy* | TTTCT | 2 |
| *LTR-Gypsy* | ATTAA | 1 |
| *LTR-Gypsy* | ACCTT | 1 |
| *LTR-Gypsy* | TTCTC | 1 |
| *LTR-Gypsy* | ATCTC | 1 |
| *LTR-Gypsy* | GCGGC | 1 |
| *LTR-Gypsy* | CTCGT | 1 |
| *LTR-Gypsy* | GTCGC | 1 |
| *LTR-Gypsy* | GTTAG | 1 |
| *LTR-Gypsy* | AACTA | 1 |
| *LTR-Gypsy* | AAGCT | 1 |
| *LTR-Gypsy* | CGAGA | 1 |
| *LTR-Gypsy* | GTCGG | 1 |
| *LTR-Gypsy* | TACTA | 1 |
| *LTR-Gypsy* | CCGGC | 1 |
| *LTR-Gypsy* | TAAGG | 1 |
| *LTR-Gypsy* | TTTAC | 1 |
| *LTR-Gypsy* | GTTTC | 1 |
| *LTR-Gypsy* | GTTAA | 1 |
| *LTR-Gypsy* | CTAAC | 1 |
| *LTR-Gypsy* | CCTAA | 1 |
| *LTR-Gypsy* | TTCCG | 1 |
| *LTR-Gypsy* | GGGGC | 1 |
| *LTR-Gypsy* | GCTAC | 1 |
| *LTR-Gypsy* | CCGGT | 1 |
| *LTR-Gypsy* | GGAGG | 1 |
| *LTR-Gypsy* | CTTCG | 1 |
| *LTR-Gypsy* | TTCCC | 2 |
| *LTR-Gypsy* | CGCAA | 1 |
| *LTR-Gypsy* | AATAC | 1 |
| *LTR-Gypsy* | TCGAA | 1 |
| *LTR-Gypsy* | GCGCC | 1 |
| *LTR-Gypsy* | TTATC | 1 |
| *LTR-Gypsy* | AGTTG | 1 |
| *LTR-Gypsy* | TAAAC | 1 |
| *LTR-Gypsy* | GATAC | 1 |
| *LTR-Gypsy* | TGCAT | 1 |
| *LTR-Gypsy* | GCCGT | 1 |
| *LTR-Gypsy* | ATCGC | 1 |
| *LTR-Gypsy* | TTAGT | 1 |
| *LTR-Gypsy* | CGCCC | 1 |
| *LTR-Gypsy* | TACAG | 1 |
| *LTR-Gypsy* | CCGAA | 2 |
| *LTR-Gypsy* | TGGCA | 1 |
| *LTR-Gypsy* | TATCT | 1 |
| *LTR-Gypsy* | GTTCC | 1 |
| *LTR-Gypsy* | TTTTG | 1 |
| *LTR-Gypsy* | CGCGC | 1 |
| *LTR-Gypsy* | TAGTAG | 1 |
| *DNA-Mutator* | GCAGCAACC | 1 |
| *DNA-Mutator* | GACTCTGGT | 1 |
| *DNA-Mutator* | TCGTCTC | 1 |
| *DNA-Mutator* | TCATGCCC | 1 |
| *DNA-Mariner* | TA | 26 |
| *DNA-Habinger* | CTC | 1 |
