# Supplementary material for: Abundance, distribution and potential impact of transposable elements in the genome of Mycosphaerella fijiensis
Source: BMC Genomics. 2012 Dec 22;13:720. doi: 10.1186/1471-2164-13-720 (PMC3562529; doi:10.1186/1471-2164-13-720)
Supplement: Additional file 2 — Sequences coding proteins downstream and upstream of full copies of the transposable elements. The table contains an analysis of the regions approximately 10,000 bp upstream and downstream of each transposable element with identification of 339 genes encoding proteins or protein domains. [file 1471-2164-13-720-S2.docx]

**Table 2 Sequences coding proteins downstream and upstream of full copies of the transposable elements**

| **Scaffold** | **Transposon** | **Gene** | **Approximate**  **distance (pb)** | | **Sequence identity (%)** | **Sequence similarity (%)** | **Access Genbank** |
| --- | --- | --- | --- | --- | --- | --- | --- |
| 1 | LTR-Gypsy | RNA binding protein | D: 5,800 | | 60 | 75 | XP_747247.1 |
| 1 | LTR-Gypsy | PX domain protein | D: 4,100 | | 63 | 78 | XP_001263638.1 |
| 1 | LTR-Gypsy | Eukaryotic translation initiation factor | U:4,000 | | 73 | 81 | XP_001938164.1 |
| 1 | LTR-Gypsy | Melibiase subfamily | U: 1,900 | | 57 | 73 | XP_747096.1 |
| 1 | LTR-Gypsy | Glutaminase A | U: 7,400 | | 65 | 77 | XP_001930459.1 |
| 1 | LTR-copia | Phospholipid-translocation P-type ATPase domain | U: 7,300 | | 72 | 85 | XP_003069255.1 |
| 1 | LTR-copia | Endonuclease XPF | U: 1,200 | | 66 | 81 | XP_001936045.1 |
| 1 | LTR-copia | ubiquitin-conjugating enzyme E2 | D: 4,933 | | 72 | 78 | XP_001593719.1 |
| 1 | LTR-Gypsy | malate synthase | D: 9,000 | | 84 | 91 | XP_001797883.1 |
| 1 | LTR_Gypsy | ELMO/CED-12 | D: 8,700 | | 51 | 67 | XP_003172626.1 |
| 1 | LTR_Gypsy | ATP-dependent helicase | U: 5,500 | | 78 | 86 | XP_001543705.1 |
| 1 | LTR_Gypsy | ABC transporter | D: 5,500 | | 70 | 83 | XP_001727592.1 |
| 1 | LTR_Gypsy | glutamate-cysteine ligase | D: 4,300 | | 77 | 86 | XP_001940223.1 |
| 1 | LTR-Gypsy | caffeine resistance | D: 7,500 | | 60 | 71 | XP_001398631.1 |
| 1 | LTR-Gypsy | DNA damage response protein | D: 8,600 | |  |  | ref\|XP_003238333.1 |
| 1 | DNA-mariner | cystathionine gamma-lyase | U: 6,800 | | 82 | 90 | XP_001398170.1 |
| 1 | DNA-mariner | adenosylhomocysteinase A | D: 9,300 | | 76 | 83 | XP_001931212.1 |
| 1 | LTR-gypsy | aminopeptidase Y | D: 7,400 | | 55 | 70 | XP_003002003.1 |
| 1 | LTR-copia | serine/threonine protein kinase | | U: 791 | 64 | 77 | XP_001819711.2 |
| 1 | LTR-copia | mannosidase MsdS | | U: 3,350 | 61 | 76 | XP_001268805.1 |
| 1 | LTR-copia | trimethyllysine dioxygenase | | U: 6,200 | 57 | 69 | XP_001938863.1 |
| 1 | LTR-Gypsy | phosphatidylinositol 3- and 4-kinase | | U: 8,000 | 52 | 70 | ref\|XP_001400633.2 |
| 1 | LTR-Gypsy | Erg-1 (putative) | | U: 7,300 | 74 | 85 | XP_001218120.1 |
| 1 | LTR-Gypsy | RBG1 | | U: 5,500 | 86 | 95 | XP_001398900.2 |
| 1 | LTR-Gypsy | MAP kinase | | D: 3,600 | 66 | 74 | XP_001930530.1 |
| 1 | LTR-Gypsy | MFS | | D: 6,800 | 58 | 65 | XP_750103.2 |
| 1 | LTR-Gypsy | RNase L | | U: 4,950 | 81 | 89 | XP_752397.1 |
| 1 | LTR-Gypsy | NADPH--cytochrome P450 | | D: 3,956 | 51 | 68 | XP_001818965.1 |
| 1 | LTR-Gypsy | sugar transporter | | D: 8,350 | 63 | 74 | XP_001823972.2 |
| 1 | LTR-Gypsy | origin recognition complex subunit Orc1 | | U: 5,500 | 53 | 64 | XP_752437.1 |
| 1 | LTR-Gypsy | Pirin | | U: 1,200 | 70 | 83 | XP_001262000.1 |
| 1 | LTR_gypsy | Prp46 | | D: 3,000 | 63 | 73 | XP_001210348.1 |
| 1 | LTR_gypsy | Importin subunit beta-4 | | U: 4,700 | 51 | 71 | XP_001825385.1 |
| 1 | LTR_gypsy | MYND domain protein | | D: 3,300 | 52 | 65 | XP_750050.1 |
| 1 | LTR_Gypsy | 2-methylcitrate synthase | | D: 4,200 | 83 | 91 | ref\|XP_965076.1 |
| 1 | LTR_Gypsy | LMBR1 domain protein | | D: 2,000 | 51 | 69 | XP_001481710.1 |
| 1 | LTR_Gypsy | 2-methylcitrate synthase | | U: 3,300 | 83 | 91 | ref\|XP_965076.1 |
| 1 | LTR_Gypsy | LMBR1 domain protein | | U: 4,800 | 51 | 69 | XP_001481710.1 |
| 1 | LTR_Gypsy | histone H3 | | D: 1,400 | 55 | 70 | XP_001525519.1 |
| 1 | LTR_Gypsy | GTP-binding protein SARA | | U:170 | 86 | 92 | XP_001930414.1 |
| 1 | LTR_Gypsy | WD40 domain | | D: 4,500 | 61 | 71 | XP_002372958.1 |
| 1 | LTR_Gypsy | histone H3 | | D: 67 | 53 | 64 | XP_001486587.1 |
| 1 | LTR_Gypsy | oxidoreductase | | U: 6,100 | 69 | 83 | XP_002847162.1 |
| 1 | LTR_Gypsy | fructose-2,6-bisphosphatase | | U: 4,800 | 73 | 83 | XP_001546391.1 |
| 1 | LTR_Gypsy | 40S ribosomal protein | | D: 6,000 | 79 | 83 | XP_001937560.1 |
| 1 | LTR_Gypsy | mRNA splicing factor-Prp17 | | D: 7,700 | 54 | 66 | XP_750623.1 |
| 1 | LTR_Gypsy | ABC transporter | | U: 7,700 | 69 | 83 | XP_750621.1 |
| 2 | LTR_Gypsy | sphingosine kinase | | D: 8,500 | 53 | 67 | XP_002629018.1 |
| 2 | LTR_Gypsy | nonribosomal peptide synthetase | | D: 7,000 | 70 | 82 | XP_002625314.1 |
| 2 | LTR_Gypsy | glycerophosphoryl diester phosphodiesterase | | U: 5,500 | 66 | 80 | YP_003882601.1 |
| 2 | LTR_Gypsy | sugar transporter family protein | | D: 2,660 | 77 | 84 | XP_001935378.1 |
| 2 | LTR_Gypsy | acetyl-CoA C-acyltransferase | | D: 7,000 | 70 | 81 | XP_001392657.1 |
| 2 | LTR_Gypsy | aspartic endopeptidase | | D: 6,200 | 53 | 61 | XP_001274608.1 |
| 2 | LTR_Gypsy | tripeptidyl-peptidase | | D: 9,000 | 58 | 72 | XP_002796821.1 |
| 2 | LTR_Gypsy | protein phosphatase 2C isoform beta | | U: 6,000 | 76 | 82 | XP_001940264.1 |
| 2 | LTR_Gypsy | nuclear protein SNF4 | | U: 9,000 | 66 | 82 | ref\|XP_001401245.2 |
| 2 | Hotspot | mitochondrial dicarboxylate transporter | | U: 3,800 | 75 | 84 | XP_001935799.1 |
| 2 | Hotspot | ribosome biogenesis protein | | D: 6,900 | 66 | 78 | XP_003234797.1 |
| 2 | LTR_Gypsy | dihydroxyacetone kinase | | D: 8,000 | 64 | 78 | ref\|XP_001940391.1 |
| 2 | LTR_Gypsy | VeA | | D: 4,700 | 56 | 70 | XP_001264513.1 |
| 2 | LTR_Gypsy | pantothenate transporter | | D: 3,500 | 55 | 65 | XP_001823283.2 |
| 2 | LTR_Gypsy | 40S ribosomal protein | | D: 8,000 | 78 | 88 | XP_001826133.1 |
| 2 | LTR_Gypsy | Vps52 / Sac2 family protein | | U: 4,300 | 50 | 67 | XP_001935416.1 |
| 2 | LTR_Gypsy | 5A-2 | | U: 3,300 | 77 | 85 | XP_001245555.1 |
| 2 | LTR_Gypsy | cytochrome P450 | | U: 4,300 | 67 | 79 | XP_001940206.1 |
| 2 | LTR_Gypsy | kinesin | | D: 8,500 | 68 | 81 | XP_002340293.1 |
| 2 | LTR_Gypsy | G proteins | | U: 4,000 | 75 | 80 | XP_001936327.1 |
| 2 | LTR_Gypsy | alpha-1,2-mannosyltransferase | | D: 6,000 | 67 | 79 | XP_001824057.2 |
| 2 | LTR_Gypsy | Sugar transporter | | D: 900 | 57 | 73 | XP_003069717.1 |
| 2 | LTR_Gypsy | alcohol dehydrogenase | | D: 9,400 | 61 | 73 | XP_001940234.1 |
| 2 | LTR_Gypsy | Histone H3 | | D: 3,500 | 51 | 65 | XP_002895320.1 |
| 2 | LTR_Gypsy | acetamidase | | D: 3,700 | 58 | 70 | XP_001940983.1 |
| 2 | LTR_Gypsy | transporter | | D: 5,500 | 50 | 71 | XP_001825827.1 |
| 2 | LTR_Gypsy | xanthine dehydrogenase | | U: 4,200 | 60 | 73 | XP_001261698.1 |
| 2 | LTR_Gypsy | pep7 | | D: 1,500 | 55 | 68 | XP_003176598.1 |
| 2 | LTR_Gypsy | Histone H3 | | D: 6,000 | 55 | 69 | NP_499608.1 |
| 2 | LTR_Gypsy | Glycosyl  hydrolase | | U: 1,500 | 61 | 74 | XP_001399199.2 |
| 2 | LTR_Gypsy | Histone H3 | | U: 6,500 | 55 | 67 | NP_595567.1 |
| 2 | LTR_Gypsy | LaeA | | D: 3,100 | 50 | 68 | XP_001827612.2 |
| 2 | LTR_Gypsy | Hsp70 family protein | | D: 6,500 | 69 | 74 | XP_001818154.2 |
| 2 | LTR_Gypsy | Ribonuclease H | | U: 3,000 | 62 | 75 | XP_001933923.1 |
| 2 | LTR_Gypsy | chitin synthase 6 | | D: 5,500 | 70 | 81 | XP_001208684.1 |
| 2 | LTR_Gypsy | chitin synthase | | U: 2,200 | 70 | 82 | XP_003071333.1 |
| 2 | LTR_Gypsy | chitin synthase | | D: 300 | 61 | 74 | XP_002626068.1 |
| 2 | LTR_Gypsy | aryl-alcohol dehydrogenase | | U: 9,500 | 66 | 78 | XP_003169338.1 |
| 2 | LTR_Gypsy | 2-nitropropane dioxygenase | | D: 2,000 | 66 | 78 | XP_001831241.1 |
| 2 | DNA-Mudr | Gel1 | | D: 1,300 | 63 | 73 | XP_001265678.1 |
| 2 | LTR_Gypsy | Histone H3 | | D: 4,800 | 55 | 68 | NP_595567.1 |
| 2 | LTR_Gypsy | Upf2 | | U: 5,800 | 65 | 81 | XP_001935341.1 |
| 2 | LTR_Gypsy | 4-hydroxythreonine-4-phosphate dehydrogenase | | D: 4,400 | 59 | 82 | YP_003689200.1 |
| 2 | LTR-copia | serine/threonine protein kinase | | D: 2,600 | 52 | 63 | XP_001398347.1 |
| 2 | LTR-copia | sphingosine N-acyltransferase | | D: 400 | 63 | 73 | XP_001820816.1 |
| 2 | LTR-copia | pif1 | | D: 700 | 52 | 64 | XP_001940398.1 |
| 2 | LTR-copia | DNA helicase | | D: 1,700 | 54 | 66 | XP_001824182.2 |
| 2 | LTR_Gypsy | feruloyl esterase | | U: 4,100 | 52 | 67 | XP_750792.1 |
| 2 | LTR_Gypsy | hippurate hydrolase | | D: 7,000 | 57 | 72 | XP_001937636.1 |
| 2 | LTR_Gypsy | FAD dependent oxidoreductase | | D: 180 | 59 | 72 | XP_001936223.1 |
| 2 | LTR_Gypsy | amidohydrolase | | U: 5,500 | 53 | 69 | XP_752490.1 |
| 2 | LTR_Gypsy | casein kinase I | | U: 3,200 | 100 | 100 | XP_366753.2 |
| 2 | LTR_Gypsy | archaerhodopsin-2 precursor | | D: 8,600 | 64 | 74 | XP_001937696.1 |
| 2 | LTR_Gypsy | Serine carboxypeptidase | | D: 190 | 50 | 65 | XP_003071795.1 |
| 2 | LTR_Gypsy | acuM | | D: 4,500 | 54 | 66 | XP_001399574.2 |
| 3 | LTR_Gypsy | SEC23 | | D: 4,000 | 81 | 89 | XP_003000127.1 |
| 3 | LTR_Gypsy | casein kinase I isoform gamma- | | D: 3,000 | 70 | 79 | XP_001934754.1 |
| 3 | LTR_copia | NudF | | D: 7,500 | 64 | 77 | XP_751175.1 |
| 3 | LTR_Gypsy | NudF | | D: 7,000 | 59 | 72 | XP_960958.2 |
| 3 | LTR_Gypsy | (Gto3) | | D: 4,500 | 60 | 72 | XP_002151056.1 |
| 3 | LTR_Gypsy | sphingomyelin phosphodiesterase | | D: 3,000 | 53 | 68 | XP_001394071.1 |
| 3 | LTR_Gypsy | transporter | | D: 4,000 | 55 | 73 | XP_001385148.1 |
| 3 | LTR_Gypsy | cell surface receptor/MFS transporter | | U: 2,800 | 55 | 71 | XP_002622254.1 |
| 3 | DNA-Mariner | lipoate-protein ligase A | | U: 500 | 64 | 75 | XP_001934570.1 |
| 3 | LTR_Gypsy | protein pucG | | D: 5,000 | 71 | 81 | XP_001934465.1 |
| 3 | LTR_Gypsy | D-galactonate dehydratase | | U: 5,000 | 74 | 82 | XP_001819482.1 |
| 3 | LTR_Gypsy | nicotinamide mononucleotide permease | | D: 7,000 | 56 | 68 | XP_572830.1 |
| 3 | LTR_Gypsy | 2-deoxy-D-gluconate 3-dehydrogenase | | D: 6,000 | 52 | 71 | XP_002622059.1 |
| 3 | DNA-Mariner | GrpB domain protein | | D: 400 | 57 | 69 | XP_001265499.1 |
| 3 | DNA-Mariner | response regulator protein | | D: 7,000 | 50 | 63 | XP_003349046.1 |
| 3 | LTR_Gypsy | SPS19 | | U: 6,000 | 62 | 77 | XP_002625253.1 |
| 3 | LTR_Gypsy | phosphoprotein phosphatase | | U: 9,000 | 95 | 99 | XP_001939922.1 |
| 3 | LTR_Gypsy | carbamoyl-phosphate synthase subunit arginine-specific small | | D: 5,000 | 79 | 88 | XP_003234973.1 |
| 3 | LTR-Gypsy | kinase-related protein | | U: 6,000 | 59 | 76 | XP_002144469.1 |
| 3 | LTR-Gypsy | short chain dehydrogenase/reductase | | U: 9,000 | 57 | 74 | XP_003177651.1 |
| 3 | DNA-Mariner | naringenin,2-oxoglutarate 3-dioxygenase | | U: 8,500 | 71 | 82 | XP_001396000.1 |
| 3 | DNA-Mariner | 3-ketoacyl-coA thiolase peroxisomal A precursor | | U: 500 | 79 | 89 | XP_751733.1 |
| 3 | DNA-Mariner | glycerol-3-phosphate O-acyltransferase | | D: 6,000 | 55 | 71 | XP_003065678.1 |
| 3 | LTR-Gypsy | D-lactate dehydrogenase | | D: 900 | 63 | 79 | XP_001827379.2 |
| 3 | LTR-Gypsy | initiation-specific alpha-1,6-mannosyltransferase | | D: 5,500 | 62 | 75 | XP_001932497.1 |
| 3 | LTR-copia | ribosome-releasing factor 2 | | U: 3,000 | 57 | 69 | XP_001823130.2 |
| 3 | LTR-copia | retromer complex subunit Vps17 | | U: 1,000 | 75 | 82 | XP_001940006.1 |
| 3 | LTR-copia | sorbitol dehydrogenase | | D: 2,000 | 59 | 72 | XP_001936306.1 |
| 3 | LTR-copia | P-loop ATPase or Walker A | | D: 8,000 | 61 | 77 | XP_003234197.1 |
| 3 | LTR-Gypsy | carboxylesterase | | U: 8,000 | 60 | 73 | XP_755184.1 |
| 3 | LTR-Gypsy | O-acetylhomoserine (thiol)-lyase | | D: 2,500 | 70 | 79 | XP_001933978.1 |
| 3 | LTR-copia | O-acetylhomoserine (thiol)-lyase | | U: 1,500 | 70 | 79 | XP_001933978.1 |
| 3 | LTR-copia | ThiJ/PfpI family protein | | D: 3,000 | 60 | 73 | XP_748199.1 |
| 3 | LTR-Gypsy | ThiJ/PfpI family protein | | U: 2,000 | 60 | 73 | XP_748199.1 |
| 3 | LTR-Gypsy | flavoprotein | | D: 8,000 | 52 | 68 | XP_001401379.2 |
| 3 | LTR-Gypsy | Glutaredoxin | | D: 9,500 | 59 | 70 | XP_003068178.1 |
| 3 | LTR-Gypsy | NADH-cytochrome b5 | | U: 8,000 | 64 | 75 | XP_001930645.1 |
| 3 | LTR-Gypsy | FAD binding domain | | U: 5,000 | 51 | 67 | XP_003070920.1 |
| 3 | LTR-Gypsy | STL1 | | U: 1,500 | 72 | 80 | XP_001933919.1 |
| 3 | LTR-Gypsy | ICE2 family | | U: 4,000 | 62 | 77 | XP_001392165.1 |
| 3 | LTR-Gypsy | alpha-1,3-glucan synthase Ags2 | | U: 1,500 | 53 | 68 | XP_755460.1 |
| 3 | LTR-Gypsy | delta-aminolevulinic acid dehydratase | | D: 5,000 | 79 | 88 | XP_961625.1 |
| 3 | LTR-Gypsy | SYF2 | | U: 8,000 | 53 | 70 | XP_002149215.1 |
| 3 | LTR-Gypsy | tyrosine protein phosphatase | | D: 6,300 | 58 | 67 | EGP89369.1 |
| 3 | LTR-Gypsy | rhamnogalacturonan acetylesterase | | D: 8,900 | 61 | 76 | XP_002378772.1 |
| 3 | LTR-Gypsy | SWIB/MDM2 domain | | D: 8,000 | 60 | 76 | EDP55545.1 |
| 3 | LTR-Gypsy | ATP synthase subunit | | U: 5,000 | 84 | 92 | EFX00799.1 |
| 3 | LTR-Gypsy | amidohydrolase | | D: 9,000 | 61 | 69 | YP_001536995.1 |
| 3 | LTR-Gypsy | suppressor of Mek1 | | U: 7,000 | 54 | 67 | XP_001939667.1 |
| 3 | LTR-Gypsy | proteasome activator subunit 4 | | D: 1,000 | 59 | 75 | XP_751700.1 |
| 3 | LTR-Gypsy | dienelactone hydrolase | | D: 7,100 | 66 | 80 | XP_746344.1 |
| 4 | DNA-MuDr | phenylalanyl-tRNA synthetase alpha chain | | U: 5,300 | 62 | 76 | XP_001941081.1 |
| 4 | DNA-MuDr | HXT11 | | U: 400 | 60 | 69 | XP_002845121.1 |
| 4 | DNA-MuDr | glutamyl-tRNA(Gln) amidotransferase | | D: 1,600 | 59 | 72 | XP_003238003.1 |
| 4 | LTR-Copia | Sly1 | | D: 7,900 | 68 | 78 | XP_754194.1 |
| 4 | LTR-Gypsy | serine family | | D: 6,400pb | 50 | 64 | XP_003194318.1 |
| 4 | LTR-Gypsy | histidinol-phosphate aminotransferase | | U: 3,500 | 64 | 75 | XP_001264347.1 |
| 4 | LTR-Gypsy | sorbitol dehydrogenase | | U: 3,000 | 74 | 84 | XP_001935119.1 |
| 4 | LTR-Gypsy | DskB -Ubiquitin-like | | U: 7,000 | 57 | 71 | XP_001393011.2 |
| 4 | LTR-Gypsy | cell wall glucanase | | D: 3,400 | 60 | 78 | XP_002624797.1 |
| 4 | DNA-Mariner | 3-phosphoinositide-dependent protein kinase 1 | | D: 9,500 | 82 | 91 | XP_001931920.1 |
| 4 | LTR-Gypsy | GTP-binding protein GUF1 | | D: 5,400 | 77 | 89 | XP_001212670.1 |
| 4 | LTR-Gypsy | oxidoreductase domain containing protein | | D: 470 | 55 | 68 | XP_001935043.1 |
| 4 | LTR-copia | oxidoreductase domain containing protein | | U: 6,600 | 55 | 68 | XP_001935043.1 |
| 4 | LTR-Gypsy | aminohydrolase | | D: 4,800 | 57 | 74 | XP_001396521.2 |
| 4 | DNA-Mariner | oxysterol binding protein 1 | | U: 4,200 | 53 | 66 | XP_001932761.1 |
| 4 | DNA-Mariner | NEDD8 conjugating enzyme (UbcL) | | U: 2,800 | 80 | 90 | XP_754183.1 |
| 4 | DNA-Mariner | MOSC domain | | U: 1,100 | 50 | 65 | XP_001267985.1 |
| 4 | LTR-Gypsy | acetamidase | | U: 9,500 | 51 | 68 | XP_746763.1 |
| 4 | LTR-Gypsy | aldehyde dehydrogenase | | D: 1,600 | 53 | 72 | XP_001398866.1 |
| 4 | LTR-Gypsy | alcohol dehydrogenase | | U: 5,600 | 64 | 80 | XP_747951.2 |
| 4 | LTR-Gypsy | Oxidoreductase | | U: 900 | 51 | 67 | XP_003068980.1 |
| 4 | LTR-Gypsy | proteasome subunit alpha type-6 | | D: 6,700 | 76 | 83 | XP_001399838.2 |
| 4 | LTR-Gypsy | NIMA-interacting protein TinC | | U: 3,900 | 54 | 65 | XP_001934824.1 |
| 4 | LTR-Gypsy | pyrazinamidase/nicotinamidase | | D: 7,500 | 53 | 63 | XP_001391911.1 |
| 5 | LTR-Gypsy | Rfc3 | | D: 5,800 | 72 | 84 | XP_001266345.1 |
| 5 | DNA-Mariner | GTP cyclohydrolase II | | U: 3,100 | 82 | 88 | XP_001931331.1 |
| 5 | LTR-Gypsy | Kem1 | | U: 7,400 | 68 | 81 | \|XP_002628504.1 |
| 5 | LTR-Gypsy | NAD dependent epimerase/dehydratase | | D: 2,600 | 50 | 64 | XP_001395715.1 |
| 5 | LTR-Gypsy | GTR1 | | U: 2,000 | 60 | 76 | XP_001941302.1 |
| 5 | LTR-Gypsy | Sodium/hydrogen exchanger | | U: 680 | 72 | 85 | XP_003069432.1 |
| 5 | LTR-Gypsy | phosphoribosylformylglycinamidine cyclo-ligase | | D: 7,600 | 64 | 77 | XP_001941001.1 |
| 5 | LTR-Gypsy | sucrose transporter | | D: 3,700 | 53 | 70 | XP_001817063.2 |
| 5 | LTR-Copia | AP-2 complex subunit mu | | D: 6,000 | 95 | 99 | XP_001941394.1 |
| 5 | LTR-Gypsy | phytanoyl-CoA dioxygenase | | U: 6,600 | 53 | 68 | XP_001940812.1 |
| 5 | LTR-Gypsy | factor mcm5 | | D: 7,700 | 71 | 80 | XP_001543371.1 |
| 5 | LTR-Gypsy | CDP-diacylglycerol-glycerol-3-phosphate 3-phosphatidyltransferase | | U: 2,800 | 61 | 76 | XP_002378393.1 |
| 5 | LTR-Gypsy | ATP-dependent bile acid permease | | D: 3,300 | 65 | 76 | XP_001931410.1 |
| 5 | LTR-Gypsy | glutathione synthetase | | D: 2,800 | 66 | 79 | XP_001931457.1 |
| 5 | LTR-Gypsy | anaphase-promoting complex | | U: 4,490 | 52 | 67 | XP_001941413 |
| 5 | LTR-Gypsy | farnesyl pyrophosphate synthetase | | U: 7,000 | 75 | 87 | XP_001941414.1 |
| 5 | LTR-Gypsy | WSC domain | | D: 3,500 | 56 | 69 | XP_003067872.1 |
| 5 | LTR-Gypsy | Histone H3 | | D: 800 | 53 | 68 | XP_001486587.1 |
| 5 | LTR-Gypsy | tRNA (cytosine-5-)-methyltransferase | | D: 140 | 62 | 73 | XP_001931558.1 |
| 5 | LTR-Gypsy | phosphoribosylformylglycinamidine synthase | | D: 7,200 | 71 | 81 | XP_001941251.1 |
| 5 | LTR-Gypsy | ankyrin | | D: 6,100 | 50 | 63 | XP_003189677.1 |
| 5 | LTR-Gypsy | HET domain | | U: 7,200 | 51 | 64 | XP_003006483.1 |
| 5 | LTR-Gypsy | GTP-binding protein 1 | | U: 6,000 | 91 | 96 | XP_002625660.1 |
| 5 | LTR-Gypsy | tetracycline transporter | | U: 2,200 | 63 | 73 | XP_002622892.1 |
| 5 | LTR-Gypsy | serine/threonine protein kinase, | | U: 2,800 | 66 | 76 | XP_001273929.1 |
| 5 | LTR-Gypsy | malate dehydrogenase | | D: 2,800 | 66 | 75 | XP_001931613.1 |
| 5 | LTR-Gypsy | short chain dehydrogenase/reductase | | U: 3,000 | 61 | 73 | XP_001931518.1 |
| 5 | LTR-Gypsy | aromatic amino acid aminotransferase 1 | | D: 6,300 | 55 | 70 | XP_001941234.1 |
| 5 | LTR-Gypsy | SNARE domain | | U: 4,600 | 62 | 77 | XP_001941286.1 |
| 5 | LTR-Gypsy | serine/threonine-protein kinase | | D: 8,115 | 51 | 63 | XP_001941280.1 |
| 5 | LTR-Gypsy | beta-glucosidase | | D: 5,800 | 51 | 63 | XP_746996.1 |
| 5 | LTR-Gypsy | vacuolar protein sorting-associated protein 45 | | D: 5,500 | 77 | 87 | XP_001931525.1 |
| 5 | LTR-Gypsy | cap binding protein | | D: 1,600 | 51 | 70 | XP_001257722.1 |
| 5 | LTR-Gypsy | mitochondrial translation optimization protein | | D: 9,000 | 62 | 72 | XP_003232605.1 |
| 5 | LTR-Gypsy | steA | | U: 4,400 | 63 | 73 | XP_001931428.1 |
| 5 | LTR-Gypsy | sugar transporter | | D: 3,000 | 67 | 80 | XP_001822181.1 |
| 5 | LTR-Gypsy | -proFAR isomerase His6 | | D: 6,800 | 53 | 64 | XP_002625789.1 |
| 6 | LTR-Gypsy | MFS transporter | | U: 7,000 | 63 | 78 | XP_001816570.1 |
| 6 | LTR-Gypsy | coatomer subunit alpha | | D: 6,000 | 73 | 85 | XP_001397559.2 |
| 6 | LTR-Gypsy | MAP kinase | | D: 6,000 | 97 | 99 | XP_001793869.1 |
| 6 | LTR-Gypsy | UDP-glucose:sterol glycosyltransferase | | D: 6,000 | 70 | 82 | XP_003065415.1 |
| 6 | LTR-Gypsy | vacuolar protease A | | U: 2,000 | 76 | 86 | XP_001819842.1 |
| 6 | LTR-Gypsy | FtsJ-like | | U: 7,000 | 54 | 70 | XP_003065132.1 |
| 6 | LTR-Gypsy | aflatoxin B1 aldehyde reductase | | D: 8,000 | 59 | 79 | XP_002845070.1 |
| 6 | LTR-Gypsy | Cupin domain | | D: 1,000 | 57 | 74 | YP_004450950.1 |
| 6 | LTR-Gypsy | aryl-alcohol dehydrogenase Aad14 | | U: 7,000 | 67 | 86 | XP_755458.1 |
| 6 | LTR-Gypsy | alcohol dehydrogenase | | D: 5,500 | 73 | 82 | XP_001825083.1 |
| 6 | LTR-Gypsy | alcohol dehydrogenase | | U: 3,500 | 73 | 82 | XP_001825083.1 |
| 6 | LTR_Gypsy | pre-mRNA splicing factor | | D: 5,800 | 66 | 79 | XP_001247216.1 |
| 6 | LTR_Gypsy | (ABC) transporter | | D: 3,500 | 61 | 75 | XP_572661.1 |
| 6 | LTR_Gypsy | SIR2 family | | D: 4,500 | 60 | 73 | XP_002144586.1 |
| 6 | LTR_Gypsy | I Sec23/Sec24 family protein | | U: 4,000 | 77 | 87 | XP_003065546.1 |
| 6 | LTR_Gypsy | 1,3-beta-glucanosyltransferase | | D: 6,000 | 52 | 71 | XP_002384844.1 |
| 6 | LTR_Gypsy | gel3 | | U: 8,000 | 52 | 71 | XP_001827615.1 |
| 6 | LTRGypsy | vacuolar protein | | U: 3,500 | 58 | 68 | XP_002623911.1 |
| 6 | LTRGypsy | ADP-ribosylation | | D: 6,500 | 53 | 56 | XP_001932338.1 |
| 6 | LTRGypsy | DNA-directed RNA polymerase I polypeptide 2 | | D: 4,800 | 67 | 79 | XP_002541550.1 |
| 6 | LTRGypsy | sodium/phosphate symporter | | D: 3,500 | 59 | 72 | XP_001817320.1 |
| 6 | LTRGypsy | sodium/phosphate symporter | | U: 4,000 | 59 | 72 | XP_001817320.1 |
| 6 | DNA-Mariner | Sec23/Sec24 family | | U: 500 | 56 | 70 | XP_001932079.1 |
| 6 | LTRGypsy | DUF803 domain membrane protein | | D: 6,500 | 78 | 89 | XP_002146486.1 |
| 6 | DNA-Mariner | golgi transport complex subunit Cog4 | | U: 4,000 | 61 | 74 | XP_001932464.1 |
| 6 | DNA-Mariner | Patatin | | U: 500 | 52 | 65 | XP_755122.1 |
| 7 | LTR-Gypsy | xylosidase precursor | | U: 7,500 | 57 | 69 | ZP_06732744.1 |
| 7 | LTR-Gypsy | sugar transporter | | D: 3,000 | 69 | 81 | XP_001821539.1 |
| 7 | LTR-Copia | DNA mismatch repair | | D: 6,700 | 63 | 79 | XP_003068089.1 |
| 7 | LTR-Gypsy | monooxygenase | | U: 3,500 | 51 | 70 | XP_001823121.2 |
| 7 | LTR-Gypsy | peptidyl-prolyl cis-trans isomerase B | | D: 9,000 | 58 | 68 | XP_001933276.1 |
| 7 | LTR-Gypsy | cyclin-dependent protein kinase | | U: 1,000pb | 55 | 66 | XP_002620584.1 |
| 7 | Hotspot | proteasome subunit alpha | | U: 4,000 | 79 | 88 | XP_001939049.1 |
| 7 | LTR-Gypsy | gamma-glutamyltranspeptidase | | D: 6,800 | 65 | 78 | XP_001933197.1 |
| 7 | DNA-MuDr | gamma-glutamyltranspeptidase | | U: 1,000 | 65 | 78 | XP_001933197.1 |
| 7 | DNA-MuDr | IZH family channel protein | | D: 400 | 57 | 62 | XP_001392002.1 |
| 7 | LTR-Gypsy | enolase/allergen Asp F 22 | | U: 5,000 | 88 | 94 | XP_002148937.1 |
| 7 | LTR-Gypsy | 26S protease regulatory subunit S10B | | U: 7,000 | 95 | 97 | XP_002627130.1 |
| 7 | Hotspot | flavin dependent monooxygenase | | D: 300 | 51 | 65 | XP_002628467.1 |
| 7 | LTR-Copia | glucose-methanol-choline (gmc) oxidoreductase | | U: 4,000 | 52 | 71 | XP_001273087.1 |
| 7 | LTR-Gypsy | alkaline serine protease | | D: 5,000 | 62 | 74 | LTR-Gypsy |
| 7 | LTR-Gypsy | Bax Inhibitor family protein | | D: 2,000 | 78 | 92 | XP_002481552.1 |
| 7 | LTR-Gypsy | histone H3 | | U: 1,300 | 54 | 68 | XP_001486587.1 |
| 7 | LTR-Gypsy | cell wall glycosyl hydrolase Dfg5 | | D: 5,000 | 51 | 65 | XP_002152839.1 |
| 8 | LTR-Gypsy | Rab GDP-dissociation inhibitor | | D: 1,000 | 71 | 80 | XP_001821971.1 |
| 8 | LTR-Gypsy | S-adenosylmethionine-dependent methyltransferase | | U: 7,000 | 52 | 68 | XP_749275.1 |
| 8 | LTR-Gypsy | aspartate aminotransferase | | U: 1,000 | 70 | 77 | XP_001933414.1 |
| 8 | LTR-Gypsy | leukotriene A-4 hydrolase | | D: 4,000 | 62 | 77 | XP_001390581.1 |
| 8 | LTR-Gypsy | amino acid transporter | | U: 4,000 | 59 | 75 | XP_001827503.1 |
| 8 | LTR-Gypsy | mitochondrial phosphate carrier protein | | U: 1,000 | 54 | 61 | XP_002487992.1 |
| 8 | LTR-Gypsy | histone H3 | | D: 4,600 | 56 | 66 | XP_760063.1 |
| 8 | LTR-Gypsy | maleylacetate reductase | | U: 4,000 | 61 | 75 | XP_001941586.1 |
| 8 | LTR-Gypsy | amidohydrolase 2 | | U: 1,000 | 50 | 66 | XP_001939855.1 |
| 8 | LTR-Gypsy | cutinase transcription factor 1 alpha | | D: 400 | 67 | 75 | XP_001400721.2 |
| 8 | LTR-Gypsy | histone H3 | | U: 4,000 | 54 | 67 | NP_595567.1 |
| 8 | LTR-Gypsy | histone cluster | | U: 1,500 | 55 | 69 | NP_001106556.1 |
| 9 | LTR-Gypsy | histone H3 | | D: 700 | 52 | 68 | XP_002743348.1 |
| 9 | LTR-Gypsy | lariat debranching enzyme | | U: 7,500 | 56 | 74 | NP_001167303.1 |
| 9 | LTR-Gypsy | extracellular lipase | | U: 4,000 | 53 | 67 | XP_001393541.2 |
| 9 | LTR-Gypsy | extracellular lipase | | U: 4,000 | 55 | 69 | XP_002627447.1 |
| 9 | LTR-Gypsy | amino acid transporter | | D: 2,000 | 63 | 77 | XP_001820491.1 |
| 9 | LTR-Gypsy | oxidoreductase, short chain dehydrogenase/reductase family | | U: 7,500 | 59 | 74 | XP_002146811.1 |
| 9 | DNA-Mariner | histone H3 | | U: 5,000 | 54 | 66 | NP_595567.1 |
| 9 | DNA-Mariner | ribose 5-phosphate isomerase A | | D: 800 | 63 | 79 | XP_003069185.1 |
| 9 | DNA-Mariner | NAD(P) transhydrogenase | | U: 6,500 | 75 | 85 | XP_002794548.1 |
| 9 | LTR-Gypsy | ribose 5-phosphate isomerase A family | | U: 9,000 | 63 | 79 | XP_003069185.1 |
| 9 | LTR-Gypsy | T-complex protein 1 subunit alpha | | D: 5,000 | 80 | 89 | XP_001939211.1 |
| 9 | LTR-Gypsy | WD40 domain | | U: 2,000 | 51 | 65 | XP_001931049.1 |
| 9 | LTR-Gypsy | FAD binding domain protein | | U: 1,000 | 64 | 79 | XP_001263972.1 |
| 9 | LTR-Gypsy | MFS transporter | | U: 7,000 | 56 | 71 | XP_001389939.2 |
| 9 | LTR-Copia | translation initiation factor eIF-2B subunit alpha | | U: 2,000 | 56 | 71 | XP_001939517.1 |
| 10 | LTR-Gypsy | phosphorylcholine phosphatase | | U: 1,000 | 61 | 73 | YP_262939.1 |
| 10 | LTR-Gypsy | histone H3 | | U: 1,000 | 51 | 66 | XP_625903.1 |
| 10 | LTR-Gypsy | acyl-protein thioesterase 1 | | D: 5,000 | 49 | 67 | XP_001933629.1 |
| 10 | LTR-Gypsy | zinc metalloproteinase | | D: 3,750 | 61 | 75 | XP_754503.2 |
| 10 | LTR-Gypsy | NADH-ubiquinone oxidoreductase | | D: 4,500 | 73 | 86 | XP_002147321.1 |
| 10 | LTR-Gypsy | (RpnI) | | D: 6,000 | 69 | 82 | XP_002147319.1 |
| 10 | LTR-Gypsy | MFS transporter | | U: 5,000 | 63 | 77 | XP_749221.1 |
| 10 | LTR-Gypsy | protein-L-isoaspartate(D-aspartate) O-methyltransferase | | U: 4,000 | 69 | 79 | XP_001933545.1 |
| 10 | LTR-Gypsy | (Scw4) | | U: 2,000 | 55 | 69 | XP_001390975.1 |
| 10 | LTR-Gypsy | Pyridoxine 4-dehydrogenase | | D: 4 | 53 | 67 | YP_004171859.1 |
| 10 | DNA-Mariner | 26S protease regulatory subunit 6B | | D: 7,000 | 94 | 97 | XP_001933672.1 |
| 10 | LTR-Gypsy | Peptidase M3 family protein | | U: 3,000 | 53 | 69 | XP_003066059.1 |
| 10 | LTR-Gypsy | glutathione transferase omega-1 | | D: 7,000 | 58 | 69 | XP_001936636.1 |
| 10 | LTR-Gypsy | mitochondrial methylglutaconyl-CoA hydratase | | U: 1,000 | 67 | 78 | XP_001542983.1 |
| 10 | LTR-Gypsy | histone H3 | | D: 1,500 | 71 | 84 | NP_595567.1 |
| 10 | Hotspot | calcium/calmodulin-dependent protein kinase | | D: 7,000 | 76 | 83 | XP_001933525.1 |
| 10 | Hotspot | sucrose-6-phosphate hydrolase | | D: 9,000 | 56 | 73 | XP_001936697.1 |
| 10 | LTR-Gypsy | oxidation resistance protein 1 | | D: 6,000 | 54 | 67 | XP_001933518.1 |
| 10 | LTR-Gypsy | molybdenum cofactor sulfurase | | U: 9,500 | 50 | 62 | XP_001401755.1 |
| 10 | LTR-Gypsy | ribonuclease H1D:800 | | D: 800 | 67 | 76 | XP_001823167.2 |
| 10 | LTR-Gypsy | phosphoglycerate mutase | | D: 7,000 | 65 | 78 | ZP_08027076.1 |
| 10 | LTR-Gypsy | histone H3 | | U: 7,000 | 54 | 68 | XP_752749.1 |
| 11 | LTR-Gypsy | apocytochrome b | | U: 500 | 67 | 87 | XP_002620055.1 |
| 11 | LTR-Gypsy | histone H3 | | U: 5,500 | 54 | 68 | XP_003329546.1 |
| 11 | LTR-Gypsy | histone H3 | | U: 800 | 68 | 85 | XP_003329546.1 |
| 12 | LTR-Gypsy | histone H3 | | U: 6,000 | 68 | 80 | XP_760063.1 |
| 12 | LTR-Gypsy | histone H3 | | U: 1 | 54 | 64 | XP_760063.1 |
| 12 | LTR-Gypsy | beta-N-acetylglucosaminidase | | U: 5,000 | 70 | 83 | XP_747213.2 |
| 12 | LTR-Gypsy | Ran exchange factor Prp20/Pim1 | | D: 2,000 | 62 | 74 | XP_746660.1 |
| 12 | LTR-Gypsy | ubiquinone biosynthesis protein | | U: 7,000 | 66 | 80 | XP_746645.1 |
| 12 | LTR-Gypsy | cytochrome C1 heme lyase | | D: 7,000 | 54 | 67 | XP_001262342.1 |
| 12 | LTR-Gypsy | HSF-type DNA-binding domain protein | | D: 2,000 | 58 | 72 | XP_001262352.1 |
| 12 | LTR-Gypsy | cytochrome C1 heme lyase | | U: 7,000 | 53 | 67 | XP_001274756.1 |
| 12 | LTR-Gypsy | (VIP1) | | D: 4,000 | 55 | 70 | XP_755336.1 |
| 12 | LTR-Gypsy | actin cytoskeleton protein (VIP1) | | U: 6,000 | 58 | 71 | XP_002148227.1 |
| 12 | DNA-MuDr | Arp8 | | U: 7,500 | 51 | 67 | XP_001821983.1 |
| 12 | LTR-Gypsy | histone H3 | | U: 3,000 | 54 | 72 | XP_752749.1 |
| 12 | LTR-Gypsy | histone H3 | | D: 500 | 53 | 66 | NP_595567.1 |
| 12 | LTR-Gypsy | histone H3 | | U: 7,000 | 53 | 66 | NP_595567.1 |
| 13 | LTR-Gypsy | histone H3 | | D: 7,000 | 55 | 68 | XP_001486587.1 |
| 13 | LTR-Gypsy | histone H3 | | U: 800 | 54 | 67 | XP_001486587.1 |
| 14 | LTR-Gypsy | histone H3 | | D: 6,000 | 53 | 66 | XP_001526473.1 |
| 14 | LTR-Copia | histone H3 | | U: 100 | 70 | 84 | XP_760063.1 |
| 15 | LTR-Gypsy | HIStone family member | | D: 1,500 | 55 | 68 | NP_499608.1 |
| 16 | LTR-Gypsy | histone H3 | | D: 4,000 | 57 | 68 | XP_760063.1 |
| 16 | LTR-Gypsy | Histone H3 | | U: 5,000 | 50 | 64 | XP_658337.1 |
| 17 | LTR-Gypsy | histone H3 | | D: 2,000 | 56 | 69 | XP_003279034.1 |
| 18 | LTR-Gypsy | histone H3 | | U: 200 | 67 | 84 | XP_003329546.1 |
| 19 | LTR-Gypsy | ubiquitin-conjugating enzyme E2 | | U: 5,000 | 62 | 73 | XP_001400652.1 |
| 19 | LTR-Gypsy | ubiquitin-conjugating enzyme E2 | | U: 8,000 | 62 | 73 | XP_001400652.1 |
| 20 | LTR-Gypsy | cytochrome oxidase subunit 2 | | U: 6,000 | 67 | 80 | YP_001648754.1 |
| 20 | LTR-Gypsy | NADH dehydrogenase subunit 1 | | U: 10,000 | 59 | 72 | YP_001648741.1 |
| 20 | LTR-Copia | NADH dehydrogenase subunit 5 | | D: 3,300 | 69 | 81 | YP_001876502.1 |
| 20 | LTR-Gypsy | histone H3 | | D: 7,300 | 72 | 87 | NP_595567.1 |

D: downstream

U: upstream
