# Supplementary material for: Abundance, distribution and potential impact of transposable elements in the genome of Mycosphaerella fijiensis
Source: BMC Genomics. 2012 Dec 22;13:720. doi: 10.1186/1471-2164-13-720 (PMC3562529; doi:10.1186/1471-2164-13-720)
Supplement: Additional file 3 — Basic structure of the Sagui transposable element. The figure contains the nucleotide sequence, the amino acid sequence, and the sequence used as a probe for the detection of Sagui in M. fijiensis populations. [file 1471-2164-13-720-S3.docx]

1. Nucleotide sequence of the *Sagui* transposable element.

CTTCC**TGTTGTAGATAAGCGGTAAACCGATAGAATTGCTTGCTATTCGAACAAAGGACTCACTAGCTACTTATATACCCTAGCGCCAAGAATCACAAGCTATTACTAAGCCGTAAGTGAATGACCTCGCAACTTCCTTCTCCTTCTCTTGCTTATAAATCCAACTAGACTCGTACATTCCCGAGTATTCTAACA**CTCCCTCTACTTAGCTATTTAGCCGTAAACCAAATAGCTAAGTCCTAATCCTTCTTAATCGTTTTTATTGTCCCTAGGACTCTTTTTACGTTTTCTTCGAACTTCCCTTTTGTTAGGGGCTTAGTAAGGAAGTCCGCGAGTTAGTCCTTTGTCGGAATATACTTTATATCGACTAGCTTGTCCTCGACTAGTTCTCGTACGTAATGTGCCGCTACGTCGATATGTTTCGTTCTAGAATGAAATTCCGGATTTCGTACTAAAGCGATAGCCGCCTTATTGTCCCCGTAGATAATCGTAGGTTCTTTATAGTCGTCTTCCGTAAGGTATCCGACTTGCTCGAGGAAATTTCGGATCCAAATAGCTTCTTTTGCGGCGTAGGATAGCGCTAAGTATTCGGCTTCCGTAGTACTAGTCGTTACGGACCTTTGTAGTTTAGTAGACCAAGATATAGGTCCTCCGGCTAGTAGGGATATATATCCCATCGTTGACTTCCTAGTTGAGTCCGCCGCGTAATCCGAATCGGTATAGATAACCGGTAGACGAGTAACTAGAGTACTCGTATTGTTTTCGTTCTTCGGGATAGGACTATACTTTATATAGTAGTCCCGAGTGTTCCCTAGGTATCTAAGGCCGTAGATACCTCCGTCGTAGTGTCTCCTAGCCGGATTTACCGTAGATTGGCTTAGTCTCTGCGTTAGAAAAGATATATCCGGCCTCGTAGCTCGTACGGTCCAATTTAGTTTTCCGATAAGTCGTTGGTATCTCCGGACGTCTACTACCTCCTCGTCCTTTGTTATAGGTAGTAAATTCGTATATCCGTTTGCCGGAGT*CGATACTCGGAAGGTTTCGTCTAGATCTAAGCCCTCTTCTCTAAGCGTATTTTCGATATAGTGCCTTTGATCTAGTAGTATAGATCCGTCGGCCTCTCTCTCGATTCGTACGCCTAGTATAAACTTAGCGGGACCTAGATCCTTCATTTCGAACCTCTGTATAAGCCCGTCCTTTAGCGGTTTAACCTCCGCCTCTGTCTTCGCTAGTATAATAAGATCGTCGACGTACAACGCGACTATTAGCGTATAGTCGCTATTAACGAAGACCGATTGATCCGCGGGTATAGCGTAGAGGTCGTATTCTTCAAAGAAGGCCGTAATAGTCTTATTCCAAACCCGTCCGGATTGTTTTAGTCCCGGTACTCCTTTTAGGAGTTTAAGTACTTTATTAGTTCCGGGAACATCTAGGCCTTCCGGGATAGCTAGGTAGACTTCCTCCGTTAGTTCTCCTAGTAGGTAGGCACTTACTACGTCTATTTGGAATATAGCAAGTCCTTTACTAGCGGCGATAGCTAGGAGAAGGCGTAGGCTTTCGAGACGTATTACCGGAGAAAACGTCTCAAAGTAATCGATTCCGTAGCGTTGCGTAAAGCCCTTTCCTACTAATCTCGCCTTAAATTTATCGATTTGTCCGTTCGGTAGT*ATTTTTACTTTAAAAACCCATTTCGGAGTAATAACGTTTGTTTGCCCTTTTCCGTCCTTCGGCTTAGGATAGTCGACAATCTCGTAGGTATTGTTAGCCGTTAAGGACTCGATGTGTTCTTTAATAGCGTCTTTCCACTGTCGGGAGTAAGGGCCGGTAATAGCCTCCGTATAGGTCTCGGGGGTAGGTATTTCGAAAGCCTTTCGATAGTCGACTTTTAGAGCTGCTAGCCCTATTTCGTCCCTTAGTCTCTTAGGTGCTTTAGGGACCCTCGTAGACCTTCGTAGTTCCCCTTCGTTTAAATCCGTAGTAGTAGAAGCCGTAGGAGTAGTAGACCTTTCGTCGTCGTTTTTATTTAAACCGTCTTCTTCGTCTTGCGGTTTCGTAGTATTATCTTCGGCTTCGGAGTCTCCCTCTACTTCCTCTACTTCTCGAGACTCTCCTTTATCTAGGATATCGATATCTCCGTCGTCGTTGTCCGTAATAGGCCGTAGGGTAGGTAGCTCTTCTCCGGATAGTAGGCTACTTCCCTTAGTCGATTCGTCGAATTTAACGCTAGTATAGATCCCGATAGCGTCCGTCTTAGGATTATAGATCCTATACTGTTTCGTAGACTTCGTATATCCGACGAAGATTCCCGGGAATGCCGTCTTTTCTAGCTTGTCTTTCCTTTTCTCTTTAGGAATAAACGGATAGGCTAGGCAGCCGAATACCCGTAAGTGTTCTCCGCTAGGCTTTCTTCCGGTATAGAGCTCCTCGGGAGTTATATCTTTTCCTTTAATAGGTAGCCTATTATGTAAATAGGTAACCGTATAGGCCGCTTCGCCCCAAAATCGTTTAGGTAATCCGGACCCTTCTAAAGCTGCCCGTACCTTAGTTATAATAGTACGGTTTTGTCTCTCTGCTACTCCGTTTTGTTCCGGAGTATAGGGCGTCGTTGTTTCGATTCGTAGTCCCGAATAGGCCTTAAGAGCTTTAATAAGCTCCGGAGCGTTGTCGATTCTTACGGCCTTTAGTTTTCTCCCGGTTTCGAGTTCGGCCTCGTTTTTCCAAGCCTTAAAGGCTTCGTATACGTCGCTTCTATAGCGAGTAAGACGGATATCTAACCGTCTCGTAGCGTCGTCCGTAATCGTTAGTATATACCTGTTTCCTCCTAAGGTACTATACTTATACGGACCCCAAAAATCGATATATACCCTATCTAGGCAATAGTCTGCCTTTTCGGGTTCTTCTCGACTTTGTCGTCTATCGGACTTCGTAGTAATACAAGTCTCGCAAGACTTATTTAACGTCGAAAGCTCCGGGACTCCTAGTAGTACTTTATATACGTTTCGTTGTTTTTCGTCTCCCGGATGTCCTAGGCGTCGGTGCCAAAGTAGCGTAGTACCTTTAGTCGTAGTAGTAGCTATTCTCGAAGTCCCGATAGACTCGGATTCCTTTAGAACGTAGGACCTTCCGACGCGTACTCCTTTTGCTAGTTCTTCGTTTCCTCTTCGAAGTATAGCCCCTTCTTTAGTAAATAGAGTCGTAATACCTCGTTCCGAGAGTTGGCTTATCGAAAGCAGGTTATAACCGATCTCCGGTACGTATATAACGTCCGGAATCGTAGTCGGAATTCCCTTAAACCGGACGAGGATTATACCGGATCCCGCGACCTTTATTGTTTTACCGTTCGCTAGAGTAACTTTTCCGGTTATTTCCTTATAGTCCGTAAAGTAGTTCTTGTCGTTAGACATATGTCTAGTGCATCCGGAGTCTAGGATCCAATCCCCCTTATTAGATAGTCGAGTCGTAGAATCGATAGCTGTCCAAGCCGCGATCTTTGATTCCTCCTTATTATTCTCTTGGATTTTTCCGTTCTTTTGGTCCCATTTATACTTCCTGCAGTCCCGTTTAAAGTGCCCGACCTTTTGGCAATAGTAACACGTCTTTCCTTTAGCTTTGTTAGGCGTATTAGTCGTATTGCCCTTCTTTCCGTTACCGGCCTTATCTTTCGCTGCTTCCTTAGTATATCGGGCTACTACTTCGTCGCCTTTAGAAGTAGTAATAGTATATTCAACGTCCCGTAGTCGCTGAACTAGTTCCTCGTAGGAAAGAGTAGATAAACCGGCCGCCTTTAAGGCGAATACGGTAGACGAATACTCTTCTCCTAAGGACGAAATAAGGACTGCCTTCTTAGAGGCCTCCGAGGGAACTTCGTCTTTATCCGCGGCCTTAATCTCGGATTGGATTTGCGTAAGAGCTGAAGCCGTAGTATCGATATCCTTAAGTGCTTTAAAGGAATAGAAACGGGAGAGTAGCGTCGAAACCCGTTCCTTTGTCGATACCTCGCTTACTTCCTTTATCTTATCGATTACCTCCTTAGTCGTACGTAGTCCTAGGAGGTGTATACGCTTATCCCCGGCCGTAGACTTAATAAACGCAACCGCTTTTGCGTCTTCTTCTTTAGCCTCTCTTGCTTTAGCCGTATCTTGGCTTATAGATTCCGGTATACCGTACCTATACCTCCAAAGGCCTTGTCCGGATAGATAGTCTTAAATTAGATCGATCCAGATAGTATAATTCTCGGTAGTAAGTTGCGGAATTCCCTTTACCGCGCCGATAGTCGTCTCGGTAGTAGACATCTTTATCGTTAGATTTCTCGACTCGTAACGGTCGTATCGTTGATATTTTTAGATCGTTTGATTCGTATTAATCGGTTCTTTTCGCCTATGGACCGGCGTCTATAGCGGAGCAATAACCGTTATCTTATTGTTGTTCGAAGTTGGTCGCTTTCTTCGATTTGCGGAGTGACGTCGGATAAGGTTTAACCTTGCGCAAGCGGGTTGCCTGATCGGGTAATTTCTCGAACTTTCTTGTTTCTTCACTTGCGTTCCGGGCTCATAAC**TGTTGTAGATAAGCGGTAAACCGATAGAATTGCTTGCTATTCGAACAAAGGACTCACTAGCTACTTATATACCCTAGCGCCAAGAATCACAAGCTATTACTAAGCCGTAAGTGAATGACCTCGCAACTTCCTTCTCCTTCTCTTGCTTATAAATCCAACTAGACTCGTACATTCCCGAGTATTCTAACA**CTTCC

1. Amino acid sequence encoded by *Sagui* transposable element.

Met S N D K N Y F T D Y K E I T G K V T L A N G K T I K V A G S G I I L V R F K G I P T T I P D V I Y V P E I G Y N L L S I S Q L S E R G I T T L F T K E G A I L R R G N E E L A K G V R V G R S **Y V L K E S E S I G T S R I A T T T T K G T T L L W H R R L G H P G D E K Q R N V Y K V L L G V P E L S T L N K S C E T C I T T K S** D R R Q S R E E P E K A D Y **C L D R V Y I D F W G P Y K Y S T L G G N R Y I L T I T D D A T R R L D I R L T R Y R S D V Y E A F K A W K N E A E L E T G R K L K A V R I D N A P E L I K A L K A Y S G L R I E T T T P Y T P E Q N G V A E R Q N R T I I T K V** R A A L E G S G L P K R F W G E A A Y T V T Y L H N R L P I K G K D I T P E E L Y T G R K P S G E H L R V F G C L A Y P F I P K E K R K D K L E K T A F P G I F V G Y T K S T K Q Y R I Y N P K T D A I G I Y T S V K F D E S T K G S S L L S G E E L P T L R P I T D N D D G D I D I L D K G E S R E V E E V E G D S E A E D N T T K P Q D E E D G L N K N D D E R S T T P T A S T T T D L N E G E L R R S T R V P K A P K R L R D E I G L A A L K V D Y R K A F E I P T P E T Y T E A I T G P Y S R Q W K D A I K E H I E S L T A **N N T Y E I V D Y P K P K D G K G Q T N V I T P K W V F K V K I L P N G Q I D K F K A R L V G K G F T Q R Y G I D Y F E T F S P V I R L E S L R L L L A I A A S K G L A I F Q I D V V S A Y L L G E L T E E V Y L A I P E G L D V P G T N K V L K L L K G V P G L K Q S G R V W N K T I T A F F E E Y D L Y A I P A D Q S V F V N S D Y T L I V A L Y V D D L I I L A K T E A E V K P L K D G L I Q R F E Met K D L G P A K F I L G V R I E R E A D G S I L L D Q R H Y I E N T L R E E G L D L D E T F R V S T P** A N G Y T N L L P I T K D E E V V D V R R Y Q R L I G K L N W T V R A T R P D I S F L T Q R L S Q S T V N P A R R H Y D G G I Y G L R Y L G N T R D Y Y I K Y S P I P K N E N N T S T L V T R L P **V I Y T D S D Y A A D S T R K S T Met G Y I S L L A G G P I S W S T K L Q R S V T T S T T E A E Y L A L S Y A A K E A I W I R N F L E Q V G Y L T E D D Y K E P T I I Y G D N K A A I A L V R N P E F H S R T K H I D V A A H Y V R E L V E D K L V D I K Y I P T K D** STOP

IN

GAG

RT

RN

**Figure1 Basic structure of the *Sagui* transposable element**. (A) The LTRs are gray with the target sites CTTCC duplicated. The sequence used as a probe is in italic and underlined. (B) ORF sequence with the RT (reverse transcriptase), RH (RNase H) and IN (integrase) domains and GAG sequence.
