# Supplementary material for: Abundance, distribution and potential impact of transposable elements in the genome of Mycosphaerella fijiensis
Source: BMC Genomics. 2012 Dec 22;13:720. doi: 10.1186/1471-2164-13-720 (PMC3562529; doi:10.1186/1471-2164-13-720)
Supplement: Additional file 4 — Alignment of four conservative sequences of Mariner elements used as probe. The figure contains the alignment of Fasta sequences used to primer design to obtain the probe for the detection of Mariner elements in M. fijiensis populations. [file 1471-2164-13-720-S4.docx]

A)

Mariner13-25 CGGTGTTTCCGAGCGAAGTTAATAAACTTAAAGAACTCGTAGCTTCTAAAGTTATCTATT 60

Mariner14-27 CGGTGTTTCCGAGCGAAGTTAATAAACTTAAAGAACTCGTAGCTTCTAAAGTTATCTATT 60

Mariner3-9 CGGTGTTTCCGAGCGAAGTTAATAAACTTAAAGAACTCGTAGCTTCTAAAGTTGTCTATT 60

Mariner14-26 CGGTGTTTCCGAGCGAAGTTAATAAACTTAAAGAACTCGTAGCTTCTAAAGTTGTCTATT 60

***************************************************** ******

Mariner13-25 ATTAGAAGGTAAAAGAGGCCTTTATTAATAGGCCTTATTAACCTCTTAAAATAGTTAATT 120

Mariner14-27 ATTATAATATAAAAGAGGCCTTTATTAATAGGCCTTATTAACCTCTTAAAATAGTTAATC 120

Mariner3-9 ATTAGAAGCCGATATTAGCCTTTATTAGTAGGCCTTATTAACCTCTCGAAGTGCTTAATC 120

Mariner14-26 ATTAGAAGCCGATATTGGCCTTTATTAGTAGGCCTTATTAACCTCTCGAAGTGCTTAATC 120

**** ** * * ********** ****************** ** * *****

Mariner13-25 TAGTAGAAGGCAAATTCGTTGTTTAAATAGCTACTATCTATTATACTAATAAGATAGTAT 180

Mariner14-27 TAGTAGAAGGCAAATTCGTCGTTTAAATAGCTACTATCTATTATACTAACAAAATAGTAT 180

Mariner3-9 TAGTAGAAGGCAAACTCGTCGTTTAAATAGCTACTATCTGTTATACTAACAAGATAGTAT 180

Mariner14-26 TAGTAGAAGGCAAACTCGTCGTTCAAATAGCTACTATCTGTTACACTAATAAGATAGTGT 180

************** **** *** *************** *** ***** ** ***** *

Mariner13-25 ACTAGCGACTAGTTAAACTAGTATTTAAGAAGGACCTTGCCTTACACTAGTAGTATTAGC 240

Mariner14-27 ACTAGCGACTAGTTAAACTAGTGTTTAAGAAGGACCTTGCCTTACACTAGTAGTATTAGC 240

Mariner3-9 ACTAGCAACTTATTAAACTAGTGTTTAAGAAGGACCTTGCCTTGTACTAGCAGCATTGGC 240

Mariner14-26 ACTAGCAACTTGTTAAACTAGTGTTTAAGACGGACCTTGCCTTATACTAGCAGTATTAGC 240

****** *** ********** ******* ************ ***** ** *** **

Mariner13-25 TTAATAAAGGCTCCGTATGCATTTACGGCTTCTACTAAGGTTATATACTTCCTATTAGTA 300

Mariner14-27 TTAATAAAGGCTCCGTAGGCATTTACGGCTTCTACTAAGGTTGTATGCTTCCTATTAGTA 300

Mariner3-9 TTAATAAAGGCTCCGTAGGCATTTACTGCTTCTACCGAGGTTATATGCTTCCTATTAGTA 300

Mariner14-26 TCAATGAAGGCTCCGTAGGCATTTACTGCTTCTACCGAGGTTATATGCTTCCTATTAGTA 300

* *** *********** ******** ******** ***** *** *************

Mariner13-25 TTAGTTGCCGCCTATAACCGCCTCTTTAGATCTATTATAACTACCTATTTAGTACCTCTA 360

Mariner14-27 TTAGTTACCGCCTATAACCGCCTCTTTAGATCTATTATAACTACCTATTCGGCACCTCTA 360

Mariner3-9 TTAGTTGCCGCCTATAACCGCCTCTTTAGATTTATTGTAACTACCTATTCGGCGCCTCTA 360

Mariner14-26 TTAGTTGCCGCCTATAACCGCCTCTTTAGATCTATTGTAACTACCTATTCGGCGCCTCTA 360

****** ************************ **** ************ * ******

Mariner13-25 ATACTAATTTAGAAACATGTCTTATCTATATTCTAGTAGTTCTCGAGACTAGTCTTATAG 420

Mariner14-27 ATACTAATCCGGAAACCTGTCTTATCTATATTCTAGTAGTTCTCGAGACTAGTCTTATAG 420

Mariner3-9 ATGCTAATCCGGAAACCTGTCTTATCTATATTCTAGTAGTTCTTAAGGCTAATCTTATAG 420

Mariner14-26 ATACTAATCCGGAAACCTGTCTTATCTATATTCTAGCAGTTCTTAAGGCTAATCTTATAG 420

** ***** ***** ******************* ****** ** *** ********

Mariner13-25 ACCTATATTAACTCGTATAGCTTATTAAACTACGAGCCGATAAACTAAGGGCCGTATACT 480

Mariner14-27 ACCTATATTAACTCGTATAGCTTATTAAACTACGAGCCGATAAGCTAAGGGTCGTATACT 480

Mariner3-9 ACCTATATTAACTCGTATAGCTTATTAAACTACGAGCCGATAAACTAAGGGTCGTATACT 480

Mariner14-26 ACCTGTATTAACTCGCATAGCTTATTAAACTACGAGCCGATAAACTAAGGGTCGTGTGCT 480

**** ********** *************************** ******* *** * **

Mariner13-25 TCCCTCCTTACTAACTCGATAGGTTTCCTTTTAATCTCTTTAATATTGTACTTCTTTATC 540

Mariner14-27 TCCCTCCTTACTAACTCGATAGGTTTCCTTTTAATCTCTTTAATATTATGCTTCTTTATC 540

Mariner3-9 TCCTTCCTTACTAACTCGATAGGCTTCCTTTTAATCTCTTTAATATTATACTTCTTTATC 540

Mariner14-26 TCCTTCCTTGCTAACTCGATAGGCTTCCTTTTAATCTCTTTAATATTATACTTCTTTATC 540

*** ***** ************* *********************** * **********

Mariner13-25 TACCTCTTATACTACTTACAACTTAATAGTCCTAGTGTTTTACCTTCTAGAAGTAACCGC 600

Mariner14-27 TACCTCTTATACTACTTACTACTTAATAATGCTAGTGTTTTACCTTCTAGAAGTAACTGC 600

Mariner3-9 TACCTTTTATACTACTTACTACTTAATAGTACTAGTATTTTACCTTCCGGAAGTAACCGC 600

Mariner14-26 TACCTCTTATGCTACTTACTACTTAATAGTGCTAGTATTTCACCTTCCGGAAGTAACCGC 600

***** **** ******** ******** * ***** *** ****** ******** **

Mariner13-25 TTTAATATTAACTCGGCCGTAGCGCGAACAAGTAACGTTAGCAATAATAAGTCTATTAAT 660

Mariner14-27 TTTAATATTAACTCGGCCGTAGCGCGAATAAGTAACGTTAGCAATAATAAGTCTATTAAT 660

Mariner3-9 TTTAATATTAACTTAGCCGTAGCGCGAATAAGTAACGTTAGTAGCGACAGGTCTATTAAT 660

Mariner14-26 TTCAATATCAACTTAGCCGTAGCGAGAATAAGTAACGTTAGCAGCGACAGGTCTATTAAT 660

** ***** **** ********* *** ************ * * * **********

Mariner13-25 TCTCGAGATCTATAGTATTCGATAAGGGCGATAGATTAGGCTTCGAAGAGGCAAGAATAC 720

Mariner14-27 TCTCGAGATCTATAGTATTTAATAAGGGCGACAAATTAGGCTTCGAAGAGGCGAGAATAC 720

Mariner3-9 TCTCGAGATCTATAGTACTCGATAAAGGCGATAGATTAGGCTTCGGAGAGGCGAGAATGC 720

Mariner14-26 TCTCGAGATCTATAGTACTCGACAAGGGCGATAGATTAGGCTTCGAAGAGGCGAGAATGC 720

***************** * * ** ***** * *********** ****** ***** *

Mariner13-25 GACGATTTACGGTCGGTACGGCTCGTATAGCTATTATATTATCGATATAATTATTAATAG 780

Mariner14-27 GACGATTTACGGTCGGTACGGCTCGTATAGCTATTATATCGTCGATATAATCGGTAATAG 780

Mariner3-9 GACGATTTACGGTCGGTACGGCTCGTATTGCTATTATATCGTCGATATAATCGATAATAG 780

Mariner14-26 GACGATTTACGGTCGGTACGGCTCGTATTGCTATTATATCGTCGATATAATCGATAATAG 780

**************************** ********** ********** ******

Mariner13-25 ATATCGTTATAGTAGTATTCGTTATAATATCGTTGTAGGTTAACTAGATAGTAGTATATC 840

Mariner14-27 ATATCGTTATAGTAGTGTTCGTTATACTATCGTTGTAGGTTAACTAGGTAGTAGTATATC 840

Mariner3-9 ATATCGTTATAGTAGTGTTCGTTATACTATCGTCGTAGGTTAACTAGGCGGTAGTATGTG 840

Mariner14-26 ATGTTATTATAGTAGTGTTCGTTACACTATCGTCGTAGGTTAACTAGGCGGTAGTGTATG 840

** * ********** ******* * ****** ************* ***** * *

Mariner13-25 TGTTTAAACTCGCGAATAGCTACTATCTAGGTAGCTACTCGGGTATCTTTCGTAAGGTTT 900

Mariner14-27 TGTTTAAACTCGCGAATAGCTACTATCTAGGCAGCTACTCGGGTATCTTTCGTAAGGTTT 900

Mariner3-9 TATTCAAACTCGCGAATTGCCGCTATCTAAGTAGCTACTCGAGTATCTTTAGTAAGGTTT 900

Mariner14-26 TGTTCAAACTCGCGAATTGCCGCTATCTAAGTAGCTACTCGAGCATCTTTAGTAAGGTTT 900

* ** ************ ** ******* * ********* * ****** *********

Mariner13-25 GTAGCCGTAGATTTATAGACGACCTTCTATACGCGTTTCTTCGACTTCCGCTTTCCT 957

Mariner14-27 ATAGCCGTAGATTTTTAGACGACCTTCCGTACGCGTTTCTTCGACTTCCGCTTTCCT 957

Mariner3-9 ATAGCTATAGATTTATAGACGGCCTTCCGTACGTATTTCTTCGACTTCCGCTTTCCT 957

Mariner14-26 GTAGCCGTGGATTTATAGACGGCCTTCCGTACGTGTTTCTTCGACTTCCGCTTTCCT 957

**** * ***** ****** ***** **** **********************

B)

>Mariner3-9

CGGTGTTTCCGAGCGAAGTTAATAAACTTAAAGAACTCGTAGCTTCTAAAGTTGTCTATTATTAGAAGCCGATATTAGCCTTTATTAGTAGGCCTTATTAACCTCTCGAAGTGCTTAATCTAGTAGAAGGCAAACTCGTCGTTTAAATAGCTACTATCTGTTATACTAACAAGATAGTATACTAGCAACTTATTAAACTAGTGTTTAAGAAGGACCTTGCCTTGTACTAGCAGCATTGGCTTAATAAAGGCTCCGTAGGCATTTACTGCTTCTACCGAGGTTATATGCTTCCTATTAGTATTAGTTGCCGCCTATAACCGCCTCTTTAGATTTATTGTAACTACCTATTCGGCGCCTCTAATGCTAATCCGGAAACCTGTCTTATCTATATTCTAGTAGTTCTTAAGGCTAATCTTATAGACCTATATTAACTCGTATAGCTTATTAAACTACGAGCCGATAAACTAAGGGTCGTATACTTCCTTCCTTACTAACTCGATAGGCTTCCTTTTAATCTCTTTAATATTATACTTCTTTATCTACCTTTTATACTACTTACTACTTAATAGTACTAGTATTTTACCTTCCGGAAGTAACCGCTTTAATATTAACTTAGCCGTAGCGCGAATAAGTAACGTTAGTAGCGACAGGTCTATTAATTCTCGAGATCTATAGTACTCGATAAAGGCGATAGATTAGGCTTCGGAGAGGCGAGAATGCGACGATTTACGGTCGGTACGGCTCGTATTGCTATTATATCGTCGATATAATCGATAATAGATATCGTTATAGTAGTGTTCGTTATACTATCGTCGTAGGTTAACTAGGCGGTAGTATGTGTATTCAAACTCGCGAATTGCCGCTATCTAAGTAGCTACTCGAGTATCTTTAGTAAGGTTTATAGCTATAGATTTATAGACGGCCTTCCGTACGTATTTCTTCGACTTCCGCTTTCCT

>Mariner13-25

CGGTGTTTCCGAGCGAAGTTAATAAACTTAAAGAACTCGTAGCTTCTAAAGTTATCTATTATTAGAAGGTAAAAGAGGCCTTTATTAATAGGCCTTATTAACCTCTTAAAATAGTTAATTTAGTAGAAGGCAAATTCGTTGTTTAAATAGCTACTATCTATTATACTAATAAGATAGTATACTAGCGACTAGTTAAACTAGTATTTAAGAAGGACCTTGCCTTACACTAGTAGTATTAGCTTAATAAAGGCTCCGTATGCATTTACGGCTTCTACTAAGGTTATATACTTCCTATTAGTATTAGTTGCCGCCTATAACCGCCTCTTTAGATCTATTATAACTACCTATTTAGTACCTCTAATACTAATTTAGAAACATGTCTTATCTATATTCTAGTAGTTCTCGAGACTAGTCTTATAGACCTATATTAACTCGTATAGCTTATTAAACTACGAGCCGATAAACTAAGGGCCGTATACTTCCCTCCTTACTAACTCGATAGGTTTCCTTTTAATCTCTTTAATATTGTACTTCTTTATCTACCTCTTATACTACTTACAACTTAATAGTCCTAGTGTTTTACCTTCTAGAAGTAACCGCTTTAATATTAACTCGGCCGTAGCGCGAACAAGTAACGTTAGCAATAATAAGTCTATTAATTCTCGAGATCTATAGTATTCGATAAGGGCGATAGATTAGGCTTCGAAGAGGCAAGAATACGACGATTTACGGTCGGTACGGCTCGTATAGCTATTATATTATCGATATAATTATTAATAGATATCGTTATAGTAGTATTCGTTATAATATCGTTGTAGGTTAACTAGATAGTAGTATATCTGTTTAAACTCGCGAATAGCTACTATCTAGGTAGCTACTCGGGTATCTTTCGTAAGGTTTGTAGCCGTAGATTTATAGACGACCTTCTATACGCGTTTCTTCGACTTCCGCTTTCCT

>Mariner14-26

CGGTGTTTCCGAGCGAAGTTAATAAACTTAAAGAACTCGTAGCTTCTAAAGTTGTCTATTATTAGAAGCCGATATTGGCCTTTATTAGTAGGCCTTATTAACCTCTCGAAGTGCTTAATCTAGTAGAAGGCAAACTCGTCGTTCAAATAGCTACTATCTGTTACACTAATAAGATAGTGTACTAGCAACTTGTTAAACTAGTGTTTAAGACGGACCTTGCCTTATACTAGCAGTATTAGCTCAATGAAGGCTCCGTAGGCATTTACTGCTTCTACCGAGGTTATATGCTTCCTATTAGTATTAGTTGCCGCCTATAACCGCCTCTTTAGATCTATTGTAACTACCTATTCGGCGCCTCTAATACTAATCCGGAAACCTGTCTTATCTATATTCTAGCAGTTCTTAAGGCTAATCTTATAGACCTGTATTAACTCGCATAGCTTATTAAACTACGAGCCGATAAACTAAGGGTCGTGTGCTTCCTTCCTTGCTAACTCGATAGGCTTCCTTTTAATCTCTTTAATATTATACTTCTTTATCTACCTCTTATGCTACTTACTACTTAATAGTGCTAGTATTTCACCTTCCGGAAGTAACCGCTTCAATATCAACTTAGCCGTAGCGAGAATAAGTAACGTTAGCAGCGACAGGTCTATTAATTCTCGAGATCTATAGTACTCGACAAGGGCGATAGATTAGGCTTCGAAGAGGCGAGAATGCGACGATTTACGGTCGGTACGGCTCGTATTGCTATTATATCGTCGATATAATCGATAATAGATGTTATTATAGTAGTGTTCGTTACACTATCGTCGTAGGTTAACTAGGCGGTAGTGTATGTGTTCAAACTCGCGAATTGCCGCTATCTAAGTAGCTACTCGAGCATCTTTAGTAAGGTTTGTAGCCGTGGATTTATAGACGGCCTTCCGTACGTGTTTCTTCGACTTCCGCTTTCCT

>Mariner14-27

CGGTGTTTCCGAGCGAAGTTAATAAACTTAAAGAACTCGTAGCTTCTAAAGTTATCTATTATTATAATATAAAAGAGGCCTTTATTAATAGGCCTTATTAACCTCTTAAAATAGTTAATCTAGTAGAAGGCAAATTCGTCGTTTAAATAGCTACTATCTATTATACTAACAAAATAGTATACTAGCGACTAGTTAAACTAGTGTTTAAGAAGGACCTTGCCTTACACTAGTAGTATTAGCTTAATAAAGGCTCCGTAGGCATTTACGGCTTCTACTAAGGTTGTATGCTTCCTATTAGTATTAGTTACCGCCTATAACCGCCTCTTTAGATCTATTATAACTACCTATTCGGCACCTCTAATACTAATCCGGAAACCTGTCTTATCTATATTCTAGTAGTTCTCGAGACTAGTCTTATAGACCTATATTAACTCGTATAGCTTATTAAACTACGAGCCGATAAGCTAAGGGTCGTATACTTCCCTCCTTACTAACTCGATAGGTTTCCTTTTAATCTCTTTAATATTATGCTTCTTTATCTACCTCTTATACTACTTACTACTTAATAATGCTAGTGTTTTACCTTCTAGAAGTAACTGCTTTAATATTAACTCGGCCGTAGCGCGAATAAGTAACGTTAGCAATAATAAGTCTATTAATTCTCGAGATCTATAGTATTTAATAAGGGCGACAAATTAGGCTTCGAAGAGGCGAGAATACGACGATTTACGGTCGGTACGGCTCGTATAGCTATTATATCGTCGATATAATCGGTAATAGATATCGTTATAGTAGTGTTCGTTATACTATCGTTGTAGGTTAACTAGGTAGTAGTATATCTGTTTAAACTCGCGAATAGCTACTATCTAGGCAGCTACTCGGGTATCTTTCGTAAGGTTTATAGCCGTAGATTTTTAGACGACCTTCCGTACGCGTTTCTTCGACTTCCGCTTTCCT

Figure 2 **Alignment of four conservative sequences of *Mariner* elements used as a probe** (A). The primers are underlined. (B) Alignment of Fasta sequences used to primer design to obtain the probe for the detection of *Mariner* element.
